# Coding transcriptome analyses reveal altered functions underlying immunotolerance of PEG-fused rat sciatic nerve allografts

**DOI:** 10.1186/s12974-020-01953-8

**Published:** 2020-10-02

**Authors:** Tyler A. Smith, Cameron L. Ghergherehchi, Haley O. Tucker, George D. Bittner

**Affiliations:** 1 Department of Molecular Biosciences, University of Texas at Austin, Austin, TX 78712 USA; 2 Department of Neuroscience, University of Texas at Austin, Austin, TX 78712 USA

**Keywords:** Axotomy, Wallerian degeneration, Polyethylene glycol (PEG), Transplantation, Allograft rejection, Nerve repair, Immune response, RNA sequencing, Transcriptome

## Abstract

**Background:**

Current methods to repair ablation-type peripheral nerve injuries (PNIs) using peripheral nerve allografts (PNAs) often result in poor functional recovery due to immunological rejection as well as to slow and inaccurate outgrowth of regenerating axonal sprouts. In contrast, ablation-type PNIs repaired by PNAs, using a multistep protocol in which one step employs the membrane fusogen polyethylene glycol (PEG), permanently restore sciatic-mediated behaviors within weeks. Axons and cells within PEG-fused PNAs remain viable, even though outbred host and donor tissues are neither immunosuppressed nor tissue matched. PEG-fused PNAs exhibit significantly reduced T cell and macrophage infiltration, expression of major histocompatibility complex I/II and consistently low apoptosis. In this study, we analyzed the coding transcriptome of PEG-fused PNAs to examine possible mechanisms underlying immunosuppression.

**Methods:**

Ablation-type sciatic PNIs in adult Sprague-Dawley rats were repaired using PNAs and a PEG-fusion protocol combined with neurorrhaphy. Electrophysiological and behavioral tests confirmed successful PEG-fusion of PNAs. RNA sequencing analyzed differential expression profiles of protein-coding genes between PEG-fused PNAs and negative control PNAs (not treated with PEG) at 14 days PO, along with unoperated control nerves. Sequencing results were validated by quantitative reverse transcription PCR (RT-qPCR), and in some cases, immunohistochemistry.

**Results:**

PEG-fused PNAs display significant downregulation of many gene transcripts associated with innate and adaptive allorejection responses. Schwann cell-associated transcripts are often upregulated, and cellular processes such as extracellular matrix remodeling and cell/tissue development are particularly enriched. Transcripts encoding several potentially immunosuppressive proteins (e.g., thrombospondins 1 and 2) also are upregulated in PEG-fused PNAs.

**Conclusions:**

This study is the first to characterize the coding transcriptome of PEG-fused PNAs and to identify possible links between alterations of the extracellular matrix and suppression of the allorejection response. The results establish an initial molecular basis to understand mechanisms underlying PEG-mediated immunosuppression.

## Background

Traumatic peripheral nerve injuries (PNIs) affect approximately 1.6% of patients who have experienced upper- or lower-limb injury in the USA and Puerto Rico, resulting in life-altering neuronal deficits [1]. Complete transection or ablation of a portion of a peripheral nerve results in immediate loss of reflex and voluntary behaviors and the Wallerian degeneration of all anucleate host axons distal to the injury site and all anucleate axons in a peripheral nerve allograft (PNA) within 1–5 days [2]. Within hours, anucleate portions of axons release damage-associated molecular patterns (DAMPs), such as high mobility group box 1 protein (HMGB1) and adenosine triphosphate (ATP), which are detected by Schwann cells and resident macrophages via toll-like receptors (TLRs) [3, 4]. This stimulation in combination with Wallerian degeneration of anucleate axons triggers Schwann cells to differentiate from a myelinating phenotype to a demyelinated phenotype [5]. Demyelinated Schwann cells release cytokines and chemokines that recruit innate immune cells such as neutrophils and hematogenous macrophages. This cytokine/chemokine release is critical to clear debris and assist Schwann cells facilitation of regenerative outgrowths from surviving proximal axons that occasionally re-innervate distal targets. In mammals, such potential re-innervation is usually a very ineffective process that can take months to complete and often results in poor restoration of lost reflex and voluntary behaviors [2].

Simple transection PNIs are typically treated by re-apposing the cut ends with epineurial microsutures (neurorrhaphy). Ablation-type injuries then are typically repaired by neurorrhaphy in combination with peripheral nerve autografts taken from a different host nerve [6]. Although autografts are currently considered the “gold standard” to repair ablation-type PNIs, autografts result in loss of donor nerve function and often produce minimal or no recovery of voluntary behaviors. Peripheral nerve allografts (PNAs) are an alternative to repair ablation-type PNIs. However, the immunogenicity of PNAs that contain living cells has severely limited their use for decades [7].

Within 7 days postoperatively, innate host immune cells responding to surgical injury recruit host T cells—the primary cells engaged in acute allorejection in the adaptive immune response [8, 9]. Endogenous antigens, often presented by donor cells via major histocompatibility complex class I (MHCI) molecules, which are expressed on all nucleated cells, are recognized by host T cells expressing the CD8 co-stimulatory receptor. Alternatively exogenous antigens, presented by MHCII molecules on either donor or host professional antigen-presenting cells, are recognized by host T cells expressing CD4 [10]. Recognition of non-self antigens and/or MHC peptides often activates acute allorejection responses in host T cells in an attempt to eliminate donor cells over a period of several weeks [7, 8]. These allorejection responses of host T cells include proliferation and differentiation into effector phenotypes, cytokine, and chemokine production to locally influence responses in nearby immune cells, killing of donor cells via perforins or granzymes, antibody-dependent cytotoxicity, and Fas receptor-mediated apoptosis. In PNAs, the primary targets of rejection are Schwann cells and endothelial cells, because of their abundance and their ability to express both MHCI and MHCII. Fibroblasts in PNAs may be also be targeted by T cells [7].

Allorejection of PNAs is commonly avoided by using decellularized allografts, immunotolerant synthetic conduits, or systemic immunosuppressants such as FK506 (Tacrolimus) that are given to the host to suppress T cell activation by inhibiting calcineurin signaling [11–14]. However, decellularized PNAs and synthetic conduits lack endogenous Schwann cells and stromal cells to support axon regeneration. Thus, immunosuppressant use can lead to opportunistic infections and liver damage. None of these techniques solve the long-existing problem of slow and ineffective restoration of nerve function after conventional neurorrhaphy.

PEG-fusion repair of singly transected sciatic PNIs in rats utilizes neurorrhaphy to non-selectively join/fuse cut axonal ends by localized application of a well-defined sequence of four pharmaceutical agents in solution, including a high concentration (50% w/w of the membrane fusogen polyethylene glycol (PEG) [15]. Compared to conventional methods, PEG-fusion substantially improves morphological, functional, and behavioral recovery after a single transection or ablation-type PNIs. Animals treated with PEG-fusion repair of single transections re-establish morphological continuity and action potential conduction across the repair site within minutes, maintain axonal integrity and innervation of neuromuscular junctions, and prevent Wallerian degeneration for many myelinated axons. Successfully PEG-fused sciatic nerves restore sciatic-mediated voluntary behaviors to near unoperated levels within 42 days post-operatively (PO) [16–18]. Unexpectedly, these effects also are observed when ablation-type sciatic PNIs are repaired using PEG-fused PNAs that are neither tissue matched nor immune suppressed. Many (40–60%) donor axons within PEG-fused PNAs do not undergo Wallerian degeneration, strongly suggesting that the axons and Schwann cells within PEG-fused PNAs are not rejected [16–18].

We previously reported [19] that innate and adaptive immune responses to PEG-fused PNAs were significantly reduced as assessed by transmission electron microscopy (TEM), immunohistochemistry (IHC), and quantitative reverse transcription PCR (RT-qPCR). Compared to negative controls (NC) that are not treated with PEG, PEG-fused PNAs at 14–21 days PO displayed significantly reduced T cell and macrophage infiltration, MHCI and MHCII expression, expression of the pro-inflammatory cytokine interferon gamma (IFN-γ) and the T cell chemoattractant C-X-C motif chemokine ligand 11 (CXCL11), as well as consistently low apoptosis [19]. Although these data strongly suggested that an immunosuppressive environment was present within PEG-fused PNAs, the underlying molecular activities associated with these effects were unknown.

Here, we describe molecular mechanisms associated with non-rejection of PNAs as assessed by coding transcriptome profiles of PEG-fused PNAs at 14 days PO—a time at which allorejection responses commonly manifest in NC PNAs [7, 20, 21]. We hypothesize that PEG-fused allografts have altered expression of genes regulating critical molecular pathways to reduce innate and adaptive inflammatory responses. We now report that 2180 gene transcripts are differentially expressed when PEG-fused PNAs are compared to negative control PNAs. Consistent with our previous immunological analyses, RNA sequencing (RNAseq) reveal that an extensive array of transcripts encoding cytokines, chemokines, transcription factors, co-stimulatory molecules, and antigen presentation machinery—each necessary for innate and adaptive allorejection responses—are significantly downregulated in PEG-fused PNAs. Many transcripts associated with Schwann cell myelinating and demyelinated states are significantly upregulated, suggesting that Schwann cells associated with both intact and degenerated axons are not rejected. We identify unanticipated links between alterations in the extracellular matrix and immunosuppression within PEG-fused PNAs. PEG-fused PNAs are particularly enriched in transcripts for extracellular matrix remodeling, cell adhesion, tissue development, fibroblast activity, and collagen production. Numerous transcripts that encode immunosuppressive proteins (e.g., thrombospondins 1 and 2, CD24, and CD276) are also upregulated.

This transcriptomic study is the first to examine the molecular details of successful PEG-fusion. It provides a crucial molecular foundation for understanding the mechanisms underlying PEG-mediated immunosuppression in PNAs, as well as in other transplanted tissue types. Clinically, PEG-fused PNAs potentially combine effective functional recovery with reduced rejection responses without decellularization or systemic immunosuppression.

## Methods

### Study design

The objective of this study was to employ RNAseq to determine significant differences in the coding transcriptome profiles between PEG-fused PNAs, negative control PNAs (operated but not treated with PEG), and unoperated control nerves. We hypothesized that differences in the immune response of these three groups should be associated with differences in gene expression profiles involved in immunotolerance/immunorejection. Because acute rejection responses to allografts in rats typically reach their peak from 14–21 days PO, PEG-fused sciatic nerve PNAs (*n* = 3 animals) and NC sciatic nerve PNAs (*n* = 3 animals) from outbred female Sprague-Dawley rats (Envigo, RRID: RGD_737903) were excised and sampled at 14 days PO (Additional File 1: Figure S1). Both treatment groups were compared to unoperated control sciatic nerves (*n* = 2 animals) as a baseline reference point for normal sciatic nerve function. DNase I-treated total RNA was extracted from each of the 8 samples and poly-A-enriched libraries were prepared and then sequenced on an Illumina next generation sequencing (NGS) platform. Initial analyses were conducted sequentially by FastQC, Tophat2, HTSeq-count, and DESeq2 software. Subsequently, analyses of gene ontology (GO) biological processes, protein families, pathways, and protein-protein interaction analyses were performed. Validation of RNAseq results for selected transcripts was performed via RT-qPCR.

For sample size, we followed the ENCODE Consortium’s best practices guidelines for performing RNAseq experiments (https://www.encodeproject.org). That is, experiments were performed with two or more biological replicates. “As part of the ENCODE pipeline, annotated transcript and genes are quantified using RSEM and the values are made available for downstream correlation analysis. Replicate concordance: the gene level quantification should have a Spearman correlation of > 0.9 between isogenic replicates and > 0.8 between anisogenic replicates.” All sample groups in this paper have an *n* = 3, except for unoperated animals that have *n* = 2. This sample number was less for unoperated animals because their inherent variation was less and COVID restrictions at UTA severely limited the acquisition of animals used for experimental trials.

### Animals

All experimental procedures were approved by standards set forth by the Institutional Animal Care and Use Committee at the University of Texas at Austin. Female Sprague-Dawley rats were housed 2–3/cage and maintained on a 12h:12h reverse light:dark cycle with food and water given ad libitum. Surgical and behavioral procedures were performed in the active cycle. Animals used for behavioral assessments were handled and trained for behavioral testing (see below) for at least 1 week prior to surgery.

### Brief description of PEG-fusion protocol

The PEG-fusion protocol (see Fig. 1 of Ghergherehchi et al. 2019 [22] for details) consists of sequential administration of four pharmaceutical agents in solution directly applied to axonal cut ends and *neurorrhaphy* (microsutures through the epi- or perineurium): (1) irrigation with 250 mM hypotonic Ca^2+^-free saline for 1–2 min to increase axoplasmic volume, open cut axonal ends, and expel intracellular membrane-bound organelles; (2) direct administration of the antioxidant methylene blue (MB) (1% in H_2_O) for 1–2 min to the opened cut ends to prevent formation of new intracellular organelles that interfere with PEG-fusion of cut ends; (3) neurorrhaphy to bring cut open ends of donor and host axons in very close apposition and to provide mechanical strength so that any PEG-fused axons within the nerve remained attached if the nerve is stretched; (4) direct application of 50% w/w 3.35 kDa PEG in distilled water (i.e., 500 mM) for 1–2 min to remove bound cell water, thereby inducing any closely apposed, open, axonal membranes to fuse (repair/join); and (5) irrigation with isotonic Ca^2+^-containing saline (290 mM) to induce vesicle formation to plug/repair/seal any axolemmal holes that may exist after PEG-induced annealing of the cytoplasm and axolemmas of open cut ends.

**Fig. 1:**
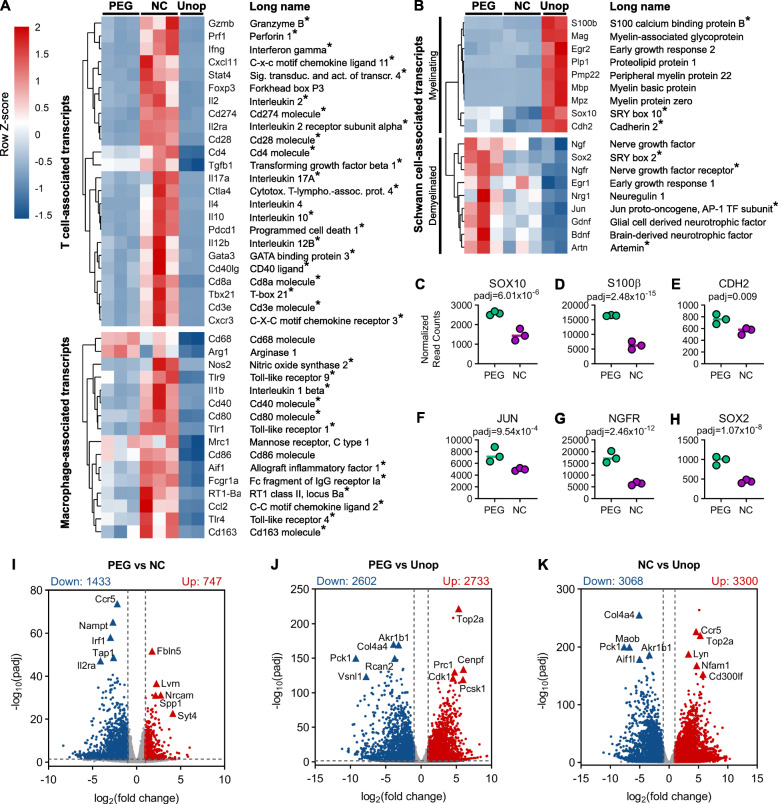
Cell-type associated transcripts and differentially expressed genes (DEGs) among PEG-fused allografts, NC allografts, and unoperated control nerves. **a** Heatmap showing relative expression of T cell-associated transcripts and macrophage-associated transcripts among all samples using normalized read counts. Each column is a sample, and each row is a gene. Transcripts are hierarchically clustered based on expression patterns across rows. Red = high expression; blue = low expression. Transcripts with statistically significant differences in expression (padj < 0.05) when compared between PEG and NC groups are marked with an asterisk. **b** Heatmap showing relative expression of Schwann cell-associated transcripts. **c**–**h** Normalized read counts of Schwann cell-associated transcripts that are significantly upregulated (PEG vs. NC, padj < 0.05). Horizontal bars indicate the mean. **i**–**k** Volcano plots showing all DEGs between **i** PEG vs. NC, **j** PEG vs. Unop, and **k** NC vs. Unop. Thresholds for log_2_ fold changes > 1 or < − 1 and padj < 0.05 were used to identify greatly upregulated (red) or downregulated (blue) DEGs for subsequent analyses. The number of up- or downregulated DEGs in each comparison is shown above each plot. Each point represents a single gene transcript. Triangles indicate notable DEGs that have very low adjusted *p* values (padj)

### Surgical procedures

Outbred female Sprague-Dawley rats weighing 225–300 g were anesthetized with inhaled 4% isoflurane/oxygen mixture (Handlebar Anesthesia) at 1.5 L/min and then maintained by a 1.5–2% mixture at 1 L/min. PEG-fusion or NC Surgeries were performed on the lateral side of the left hindlimb. The right hindlimb served as an intact control.

A 2–3.5 cm incision was made through the skin and the left biceps femoris muscle to expose the sciatic nerve. Connective tissue around the sciatic nerve was trimmed with microscissors. Complete sciatic nerve transections were made in calcium-containing isotonic extracellular fluid and/or sterile isotonic Lactated Ringers (Dechra) by fine dissection scissors to completely sever all axons as well as their endo-, peri-, and epineural sheaths. A 6–8 mm segment was ablated in mid-thigh, leaving an 8–10 mm gap between cut axonal ends in the proximal and distal stumps of the host nerve. Because intact nerves were under tension, an ablation produces a gap that was several mm longer than the removed segment. For PNAs, a donor PNA that matched the diameter of the host PNA was obtained from the left or right sciatic nerve of another wild-type Sprague-Dawley rat that was neither tissue matched nor immune-suppressed in donor or host. Donor PNAs were 1–3 mm longer than the gap created by the ablated segment of the host nerve and stored in calcium-free, hypotonic saline (Plasmalyte A (Baxter)) at 2 °C for 30 min to 6 h before use. Identical procedures for neurorrhaphy and PEG-fusion were performed for the proximal and distal ends of all PNA co-optation sites.

For all PEG-fused and NC groups, the PNAs and host sciatic nerves were washed with hypotonic Plasmalyte A and 1% methylene blue (MB) (Acros Organics). All axonal ends were carefully trimmed to provide smooth cut ends whose flat planes can be very closely apposed with at least four 10-0 microsutures through the epineurium. Nerves that were to be PEG-fused received a sterile hypotonic solution of 50% w/w 3.35 kDa PEG (Sigma Aldrich) in distilled water directly applied for 1–2 min to the lesion sites to non-specifically repair/fuse closely apposed cut axonal ends. After neurorrhaphy, lesion sites of PEG-fused and NC PNAs were washed several times with sterile isotonic Lactated Ringers containing calcium to repair any remaining axolemmal holes with calcium-induced vesicles or other membrane-bound structures. As previously described [16–19, 22], compound action potentials (CAPs) and/or compound muscle action potentials (CMAPs) were elicited before severing any nerves, and again after PEG-fusion of PNAs by stimulating proximal to all lesion sites to insure that the procedure was successful. CAPs and CMAPs were not elicitable after nerve repair in negative control PNAs. Muscle incisions were closed with 5-0 sutures and the skin was closed with wound clips. Animals recovered from surgery on heated pads and were returned to standard housing. Animals to be tested for behavioral recovery received a 5 mg/kg subcutaneous injection of carprofen (Putney, Inc.).

### RNA extraction

After sacrificing the animals using potassium chloride under anesthesia, unoperated control nerve segments (*n =* 2), 14 days PO PEG-fused PNAs (*n =* 3), and 14 days PO NC allografts (*n =* 3) were immediately excised, sliced into 0.5 mm pieces with a scalpel blade, and stored in RNAlater (Invitrogen) overnight at 4 °C to prevent RNA degradation. The tissue was then placed onto a petri dish with TRIzol (Ambion) and minced into smaller pieces with scissors. The minced tissue along with the TRIzol was then transferred to a dounce homogenizer and ground until the previously white tissue became nearly transparent. Chloroform was added to the solution, the solution was centrifuged, and the extracted aqueous layer was then combined with an equal volume of 100% ethanol before transferring it to a RNeasy Mini kit (QIAGEN) spin column (including RNase-free DNase I digestion) for RNA extraction. Total RNA concentration and purity was initially quantified with a Nanodrop 1000 spectrophotometer (Thermo Scientific, RRID:SCR_016517), and the RNA integrity was determined via BioAnalyzer 2100 (Agilent Technologies, RRID:SCR_018043). All RNA used for library preparation and RT-qPCR had RNA Integrity Numbers (RIN) between 7.1 and 8.2.

### RNAseq and bioinformatics analyses

Prior to library preparation, RNA concentration was quantified once again with a Qubit fluorimeter (ThermoFisher). Library preparation, sequencing, and initial bioinformatics analyses to determine differential expression were performed at University of Texas MD Anderson Science Park (MDACC; Smithville, TX). Libraries for each of 8 independent samples were generated with 0.13–4 μg of total RNA per sample using a TruSeq Stranded mRNA Library Prep kit (Illumina) according to manufacturer’s instructions. Libraries were sequenced on a single lane of a HiSeq 3000 unit (Illumina, RRID: SCR_016386) to collect a minimum of 20 × 10^6^ 75 bp paired-end reads per sample. Quality control of raw reads was performed using FastQC (RRID:SCR_014583). Tophat2 was used for mapping and alignment of reads to the reference genome for *Rattus norvegicus* (Rnor 6.0). Mapping rates ranged from 93.2 to 95%, while alignment rates ranged from 84.1 to 89.4% (Additional File 2: Table S1).

Read counts were then generated with HTSeq-count (RRID:SCR_011867). The DESeq2 Bioconductor package (RRID:SCR_015687) was used to generate normalized counts using the median of ratios normalization method [23] to perform differential expression analyses. Hierarchical clustering of normalized reads and heatmap generation was performed using the “pheatmap” R package (RRID:SCR_016418) on normalized read counts. Correlation matrices were created using the “pheatmap” R package combined with the “cormat” R function. Principle component analysis (PCA) of normalized read counts between samples was performed using the “ggfortify” R package. Volcano plots of differentially expressed genes (DEGs) were generated using Graphpad Prism 8 software (RRID:SCR_002798).

Our selection criteria for DEGs were transcripts that had log_2_ fold changes > 1 or < − 1 and adjusted *p* values (padj) < 0.05. Visualized networks of GO annotations for biological processes were generated using BiNGO 3.0.4 combined with Cytoscape 3.7 software (RRID:SCR_005736; RRID:SCR_003032) [24, 25]. We employed KEGG (Kyoto Encyclopedia of Genes and Genomes) pathway annotations (https://www.genome.jp/kegg) (RRID:SCR_012773) [26] and InterPro protein family annotations (https://www.ebi.ac.uk/interpro) (RRID:SCR_006695) [27], generated using DAVID 6.8 (Database for Annotation, Visualization, and Integrated Discovery (https://david.ncifcrf.gov)) (RRID:SCR_001881) [28]. Protein-protein interaction networks for all DEGs were analyzed with the STRING protein database (https://string-db.org) (RRID:SCR_005223) [29] combined with Cytoscape 3.7 software. STRING networks were generated using the default confidence threshold of 0.4 (medium confidence).

### Quantitative reverse transcription PCR

For PEG-fused and NC PNA samples, at least 200 ng of RNA per sample was reverse transcribed into cDNA using a High-Capacity cDNA Reverse Transcription Kit (Applied Biosystems). For unoperated control nerves, which contained lower amounts of RNA, 40 ng of RNA per sample was used. Samples were run on a PTC-200 thermocycler (MJ Research) according to guidelines provided by the kit manufacturer. Then, 1 ng of cDNA and 500 nM of each primer per 20 μl reaction was used for quantitative PCR. Primers were designed via the PrimerQuest tool by Integrated DNA Technologies (IDT) using complimentary mRNA transcript sequences derived from National Center for Biotechnology Information (NCBI) GenBank databases for *Rattus norvegicus* (www.ncbi.nlm.nih.gov/genbank) (Table 1). qPCR reactions were prepared using PowerUp SYBR Green Master Mix (Applied Biosystems) and run for 40 cycles in triplicate on a ViiA7 qPCR thermocycler (Applied Biosystems) in a 96-well plate according to guidelines provided by the kit manufacturer. Glyceraldehyde 3-phosphate dehydrogenase (GAPDH) was used as a reference gene to normalize the expression data from other transcripts. RNA transcript expression for each gene, displayed as fold changes over unoperated control nerves, was quantified using the ΔΔCt method of relative quantification [30].

**Table 1:** Primer sequences used for RT-qPCR

Gene transcript	Accession number	Forward primer (5′-3′)	Reverse primer (5′-3′)
*Shh*	NM_017221.1	CTGGATTCGACTGGGTCTACTA	GGAAGCAGCCGTCAGATTT
*Spp1*	NM_012881.2	CACCAAGGACCAACTACAA	TGCCAAACTCAGCCACTT
*Sox8*	NM_001106989.1	CCCATGGTGAAAGCATGAAAG	TGGGAAAGACCTGTGGTAATG
*Fbln5*	NM_019153.3	CCTACTCCAATCCCTACTCTACA	TACCCAAAGCGACAGACAAG
*Cd24*	NM_012752.3	CTTGCCCATTCTGGGATCTAAT	GTTCCCGGGTAGGTTTCTAAAG
*Ngfr*	NM_012610.2	TCTGGCCAAAGAAGAGGATTAC	CATCCTGTGTGTGAGAGAGAAG
*Col8a1*	NM_001107100.1	CTCTACAGCTGCTGGGAATAC	GTGGTATCTGAGGAGGGATTTG
*Ctsd*	NM_134334.2	CACATCCTTCGACATCCACTAC	TCCACCTTGATACCTCCTAAGT
*Thbs1*	NM_001013062.1	ACTGAGAGGATGACGACTATG	GTAGGACTGGGTGACTTGTTTC
*Thbs2*	NM_001169138.1	CCCAGAGGCAGTTTGAGATT	CATCCTCCAGGAAGTTGGTATG
*Icam1*	NM_012967.1	GTATCCATCCATCCCACAGAAG	CAGTTGTGTCCACTCGATAGTT
*Ccr5*	NM_053960.3	GCTAGGCAGAGGAGAATGTTAG	TGTCTCCTCCTCCCAGTAAA
*Ccl5*	NM_031116.3	CAGAGAAGAAGTGGGTTCAAGA	GAGCAAGCAATGACAGGAAAG
*Irf1*	NM_012591.1	CTCACCAAGAACCAGAGGAAAG	AGATAAGGTGTCAGGGCTAGAA
*Gzmb*	NM_138517.3	AACCAGGAGATGTGTGCTATG	CCTCTTGTAGTGTGTCTGAGTATTT
*Faslg*	NM_012908.1	GGTGCTAATGGAGGAGAAGAAG	TAAATGGTCAGCAACGGTAAGA
*Il10*	NM_012854.2	AGTGGAGCAGGTGAAGAATG	GAGTGTCACGTAGGCTTCTATG
*Nos2*	NM_012611.3	TGGAGCGAGTTGTGGATTG	CCTCTTGTCTTTGACCCAGTAG
*Cxcl11*	NM_182952.2	GTGCCCTGCAAACATTTCTAC	GTGGGAAGCCAGTGTGATTA
*Ifng*	NM_138880.2	CGAATCGCACCTGATCACTAA	TGGATCTGTGGGTTGTTCAC

### Immunohistochemistry

Methods for immunohistochemistry and immunostaining quantification in sciatic nerve tissue were described in detail previously [19]. Briefly, nerve segments 1–2 cm in length were excised and fresh-frozen in OCT (Electron Microscopy Sciences) with liquid nitrogen. For PNA treatment groups, nerve grafts were trimmed with a scalpel 1–2 mm from the suture line on the graft tissue side so that proximal or distal segment tissue was not included. Then, 6-μm transverse sections were cut on a Cryostar NX50 Cryostat (Thermo Scientific). Slides were air-dried for 30 min before fixing with acetone for 5 min. Tissue sections were then washed twice with PBS for 5 min each before blocking with goat serum solution (10% goat serum/0.1% Triton X-100/0.01% Sodium azide) for 15 min. Primary antibody dilutions prepared in goat serum solution were incubated on the slides either overnight at 4 °C or for 1 h at room temperature. We used primary antibodies against rat COL1A1 (1/200; Cat# 7-2C12; mouse monoclonal; DSHB; RRID not available), rat CD24 (1/50; Cat# 10600-1-AP; rabbit polyclonal; Proteintech; RRID: AB_10646440), rat THBS1 (1/200; Cat# LS-C137099; rabbit polyclonal; LifeSpan Biosciences; RRID: AB_10947502), rat THBS2 (1/100; Cat# PA5-97117; rabbit polyclonal; Invitrogen; RRID: AB_2808919), and rat CD276 (1/100; Cat# sc-376769; rabbit polyclonal; Santa Cruz Biotechnology; RRID not available). Tissue sections were washed three times with PBS for 5 min each, then incubated with secondary antibodies prepared in goat serum solution for 30 min, protected from light. Secondary antibodies included anti-mouse Alexa Fluor 488 (1/1000; Cat# R37120; goat polyclonal; Invitrogen; RRID: AB_2556548) and anti-rabbit Alexa Fluor 594 (1/500; Cat# R37117; goat polyclonal; Invitrogen; RRID: AB_2556545). This was followed by three more washes with PBS for 5 min each, addition of one drop of 4′,6-diamidino-2-phenylindole (DAPI) counterstain (IHC-Tek) to each slide to label nuclei, and two more washes with PBS for 5 min each. Slides were mounted with Fluoromount-G (Invitrogen). Fluorescent images were acquired using a × 40 objective (oil-immersion lens) on an Axiovert 200M fluorescent light microscope (Zeiss) equipped with an Axiocam HR3 camera (Zeiss). FIJI software (RRID: SCR_002285) was used to analyze all images.

### Statistical analyses

For RNAseq, differential expression among treatment groups and statistical analyses were performed using DESeq2 to generate negative binomial linear models and the Wald Chi-Squared Test. *P* values were adjusted with Benjamini-Hochberg correction. Transcripts with adjusted *p* values (padj)<0.05 were considered to be statistically significant in each comparison between treatment groups. Statistical analyses of GO annotation enrichment via BiNGO was performed using the Hypergeometric test and Benjamini and Hochberg false discovery rate correction; threshold padj < 0.05. Statistical analyses of KEGG and InterPro annotation enrichment via DAVID was performed using the Fisher Exact test, which determines whether the proportions of transcripts falling into each annotation category differs among groups; threshold *p* value < 0.05. Comparisons of log fold changes for selected transcripts between RNAseq and RT-qPCR were made in Graphpad Prism 8 software (RRID: SCR_002798), using the means and standard errors for each transcript. Correlation analyses of these selected transcripts were performed in Graphpad Prism 8 using parametric Pearson correlation analysis and linear regression. For immunohistochemical analyses, comparisons of means and standard deviations were analyzed between PEG-fused PNAs, NC PNAs, and unoperated control nerves using one-way ANOVA, followed by Tukey’s multiple comparisons tests in Graphpad Prism 8*.* No data points or animal subjects used in this study were omitted; any outliers are included in each analysis.

## Results

### T cell and macrophage-associated transcripts are downregulated and Schwann cell-associated transcripts are upregulated in PEG-fused PNAs

We previously demonstrated through morphological and IHC analyses that PEG-fused PNAs were significantly reduced in T cell and macrophage infiltration and that these tissues contained numerous intact, large-diameter axons that were still myelinated by accompanying Schwann cells [19]. However, we had not yet investigated these cell types within PEG-fused PNAs for transcriptional profiles that may underlie particular activation states or cell subtypes.

To better understand the overall variance in normalized read counts (Additional File 3: Fig. S2A) for all transcripts among treatment groups and among individual samples, we performed Pearson correlation analyses and principle component analyses (PCA) (Additional File 3: Figs. S2B, S2C). PCA indicated that individual samples within each treatment group clustered together, had similar expression profiles with no strong outliers, and the treatment groups themselves were biologically distinct from one another (Additional File 3: Fig. S2B). Strong sample-to-sample correlations in expression profiles were found within treatment groups, with PEG and NC groups showing greater correlation with each other than with the unoperated control group (Additional File 3: Fig. S2C).

We then examined the normalized read counts for transcripts commonly expressed or associated with either T cells, macrophages, or Schwann cells and compared their expression patterns among treatment groups via heatmaps (Fig. 1a, b). Transcripts with statistically significant differences in expression (padj < 0.05) compared between PEG and NC groups were marked with an asterisk. As shown in Fig. 1a, most transcripts associated with T cells and/or macrophages were downregulated in PEG-fused PNAs compared to NC PNAs. Downregulated transcripts included T helper 1 (Th1)-associated proinflammatory cytokines interleukin 2 (IL2), interleukin 12B (IL12B), interferon gamma (IFNG), and cytotoxic effectors that induce apoptosis in target cells, including Perforin (PRF1) and Granzyme B (GZMB) [8, 31–33]. Notable are the cytokine interleukin 4 (IL4), produced by Th2 cells; interleukin 17A (IL17A), produced by pro-inflammatory Th17 cells (34); as well as Interleukin 10 (IL10) and transforming growth factor beta 1 (TGFB1), produced by immunosuppressive (FOXP3)^+^ T regulatory T cells (Tregs) [34], which were downregulated in PEG-fused PNAs compared to NC PNAs. The transcription of co-stimulatory receptors (CD3, CD8, CD4, CD28, CD40LG), co-inhibitory receptors such as cytotoxic T-lymphocyte-associated protein 4 (CTLA4) and programmed cell death 1 (PDCD1), and downstream transcription factors that drive T cell activation such as GATA binding protein 3 (GATA3) [10, 35] were downregulated in PEG-fused PNAs relative to NC PNAs. Although each of these representative transcripts were downregulated in PEG-fused PNAs relative to NC PNAs, they were significantly upregulated in PEG-fused PNAS relative to Unoperated Control nerves (Additional File 4: Table S2).

Likewise, macrophage-associated profiles revealed significant downregulation of numerous inflammatory “M1” macrophage-associated transcripts, including (1) the pro-inflammatory cytokine interleukin 1 beta (IL1B); (2) and nitric oxide synthase (NOS2) which is required for the respiratory burst attack response; and (3) toll-like receptors 1, 4, and 9 (TLR1/4/9), which are critical for initiating innate immune responses against damaged or pathogen-associated molecules [36]. On the other hand, two transcripts commonly associated with the anti-inflammatory “M2” macrophage state, arginase 1 (ARG1) and mannose receptor C type I (MRC1/ CD206), were highly expressed in PEG-fused PNAs, although not to a statistically significant degree when compared to NCs. Proteins encoded by each of these transcripts contribute to tissue repair and remodeling functions. We also found that expression of CD68, which encodes a lysosomal protein involved in phagocytosis [37], was upregulated in PEG-fused PNAs. This finding was unanticipated based on our previous IHC analyses in which CD68 immunostaining in 14d PO PEG-fused PNAs was significantly decreased [19]. As with T cell-associated transcripts, macrophage-associated transcripts were significantly upregulated in PEG-fused PNAs relative to unoperated control nerves (Additional File 4: Table S2).

Lastly, the expression of genes associated with the myelinating Schwann cell phenotype, including SRY-box 10 (SOX10), myelin basic protein (MBP), and myelin-associated glycoprotein (MAG) [5], were most highly expressed in unoperated control nerves (Fig. 1b). PEG-fused PNAs displayed upregulation of numerous transcripts associated with the demyelinated repair Schwann cell phenotype, which is triggered upon response to axonal injury. Examples include the AP-1 transcription factor subunit JUN, SRY-box 2 (SOX2), nerve growth factor receptor (NGFR), and glial-derived neurotrophic factor (GDNF). Several additional transcripts associated with both myelinating and demyelinated Schwann cell phenotypes were significantly upregulated as well in PEG-fused PNAs compared to NC PNAs (Fig. 1c–h).

Overall, these results suggested that pro-inflammatory T cell and macrophage-mediated activities were significantly reduced in PEG-fused PNAs, and that PEG-fused PNAs contained mixed populations of myelinating and demyelinated Schwann cell phenotypes. To identify all differentially expressed genes (DEGs) among treatment groups for employment in functional annotation and enrichment analyses, we next used threshold criteria for log_2_ fold change and adjusted *p* value.

### Highly downregulated and upregulated transcripts in PEG-fused PNAs

In this study, we defined the criteria for a DEG as having a log_2_ fold change in expression of > 1 or < − 1 and an adjusted *p* value (padj) < 0.05. Volcano plots were employed to visualize these threshold criteria as applied to all transcripts when comparing PEG vs. NC, PEG vs. Unop, and NC vs. Unop (Fig. 1i–k). PEG vs. NC comparisons (Fig. 1i) yielded 1433 downregulated DEGs and 747 upregulated DEGs.

The five most downregulated transcripts (ranked by padj) were involved in chemokine/cytokine signaling and antigen presentation to T cells and myeloid cells. These include C-X-C motif chemokine receptor 5 (CCR5), interferon regulatory factor 1 (IRF1), transporter 1, ATP binding cassette subfamily B member (TAP1), and interleukin 2 receptor alpha (IL2RA) [10]. Nicotinamide phosphoribosyltransferase (NAMPT), the rate-limiting component within the NAD synthesis pathway and an essential factor in lymphocyte survival [38], also was significantly downregulated. The top 5 upregulated transcripts in the PEG group consisted of Fibulin 5 (FBLN5), an integrin-binding matricellular protein that is upregulated during tissue injury and involved in endothelial cell adhesion [39]; Laeverin (LVRN), an amino peptidase usually found in trophoblasts [40]; neuronal cell adhesion molecular (NRCAM), involved in directional signaling during axonal cone growth [41]; and secreted phosphoprotein 1 (SPP1), a matricellular protein that regulates tissue remodeling and cytokine production [42]. Both PEG vs. Unop and NC vs. Unop comparisons (Fig. 1j, k) showed downregulation of cell metabolic regulators aldo-keto reductase family 1 member B (AKR1B1) and phosphoenolpyruvate carboxykinase 1 (PCK1), as well as the collagen subunit collagen type IV alpha 4 chain (COL4A4).

The five most upregulated transcripts from the PEG vs. Unop comparison included primarily cell cycle progression mediators such as topoisomerase 2A (TOP2A), centromere Protein F (CENPF), and cyclin-dependent kinase 1 (CDK1) [43]. The top 5 upregulated transcripts from the NC vs. Unop comparison primarily were composed of cytokine response modulators, such as CCR5, LYN proto-oncogene, Src family tyrosine kinase (LYN), and NFAT activating protein with ITAM motif 1 (NFAM1) [36]. A complete listing of all normalized read counts and DEGs for all treatment group comparisons can be found in (Additional File 4: Table S2).

These results collectively suggested that PEG-fused PNAs may be enriched in extracellular matrix remodeling, cell adhesion, and/or cell cycle regulation processes. This interpretation of the DEG data was thereafter validated via gene ontology analyses.

### Transcripts for extracellular matrix remodeling, cell adhesion, and tissue development processes are enriched in PEG-fused PNAs

**Fig. 2:**
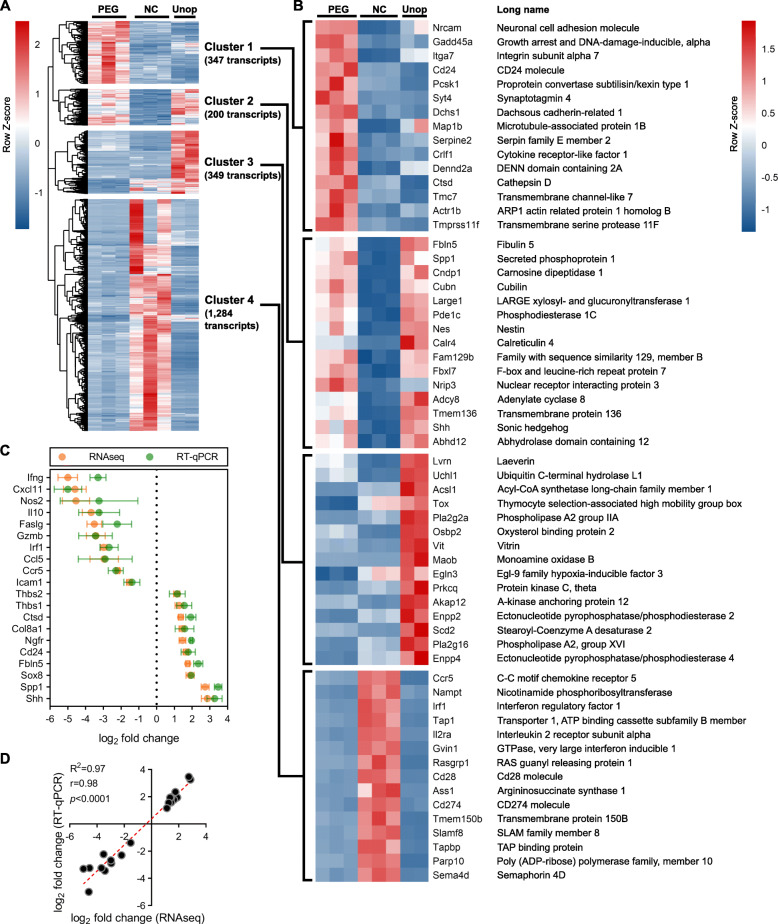
Division of DEGs into clusters based on expression patterns among treatment groups; validation of RNAseq results. **a** Heatmap showing relative expression of transcripts based on normalized read counts for all DEGs with log_2_ fold changes > 1 or < − 1 and padj < 0.05 among PEG and NC groups (2180 DEGs total). Red = high expression; blue=low expression. The heatmap is split into 4 distinct clusters of expression patterns among treatment groups, based on k-means clustering: cluster 1 (highest expression in PEG), cluster 2 (high expression in both PEG and Unop), cluster 3 (highest expression in Unop), and cluster 4 (highest expression in NC). **b** Heatmaps showing the top 15 DEGs in each cluster, ranked by padj (PEG vs NC comparison). **c** Comparison of log_2_ fold changes between RNAseq and RT-qPCR for 10 upregulated and 10 downregulated transcripts of interest (comparing PEG vs NC) that are represented in clusters 1, 2, or 4. Data represents the mean ± SEM (*n* = 3 animals per treatment group). **d** Correlation of RNAseq and RT-qPCR results from (**c**), using linear regression and Pearson correlation analyses. Each point indicates a specific transcript

In order to determine which categories of biological processes and cellular pathways were enriched in each treatment group, we created a hierarchically clustered heatmap of all DEGs from the PEG vs. NC comparison (2180 DEGs total) (Fig. 2a). We divided the heatmap into four distinct clusters, based on k-means clustering of expression patterns between each treatment group. From each cluster, we were then able to identify DEGs that were most highly expressed in the PEG group (cluster 1; 347 transcripts), highly expressed in both PEG and in Unop (cluster 2; 200 transcripts), most highly expressed in Unop (cluster 3; 349 transcripts), and most highly expressed in NC (cluster 4; 1,284 transcripts) (Fig. 2a, b) (Additional File 5: Table S3). The transcript IDs were extracted from each cluster and entered into BiNGO. This allowed us to derive functional annotations for biological processes via GO. We then validated the differential expression of 20 selected transcripts represented in clusters 1, 2, and 4 via RT-qPCR (total of 10 upregulated transcripts and 10 downregulated transcripts) (Fig. 2c). The log_2_ fold change measurements for each gene transcript assayed via RT-qPCR closely approximated those derived via RNAseq. These results were confirmed by correlation analyses for all 20 transcripts that yielded an *R*^2^ of 0.97, Pearson correlation coefficient *r* of 0.98, and *p* < 0.0001 (Fig. 2d).

**Fig. 3:**
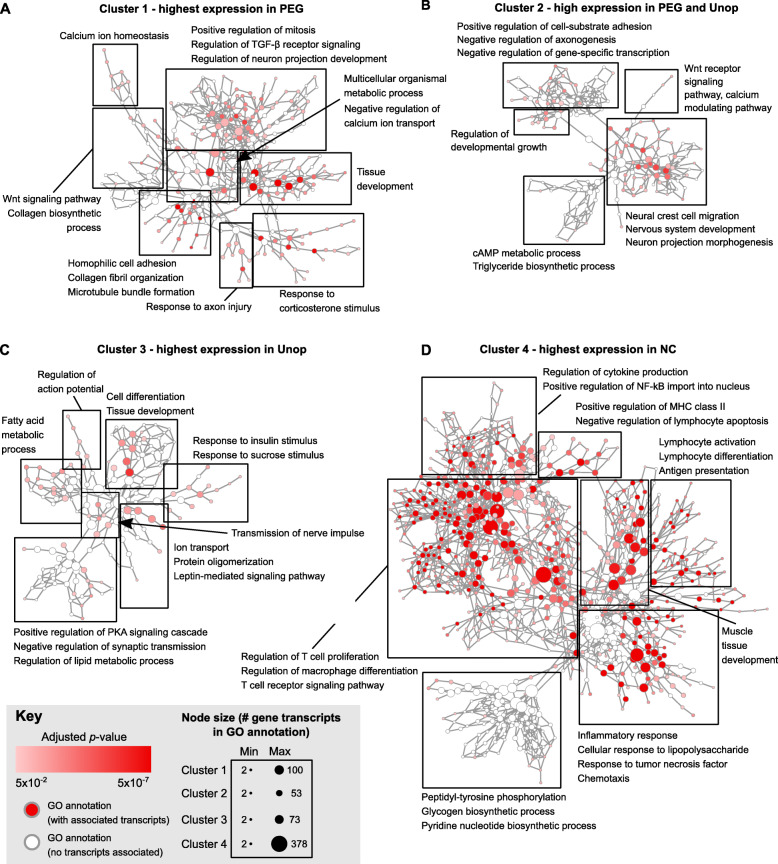
Visualization of overrepresented biological processes in clusters 1–4 using BiNGO for gene ontology (GO) analysis. Each node in the networks for **a** cluster 1, **b** cluster 2, **c** cluster 3, and **d** cluster 4 represents a single GO annotation for an overrepresented biological process. The size of the node corresponds to the number of transcripts associated with the GO annotation, while the color shade of the node indicates the padj of each GO annotation (hypergeometric test; Benjamini and Hochberg false discovery rate correction; threshold padj < 0.05). Deeper color = lower padj. Blank nodes do not contain transcripts associated with clusters 1–4. Networks are organized hierarchically from broader “parent” terms containing large numbers of transcripts (e.g., “cellular process”) to more specific “child” terms containing fewer numbers of transcripts (e.g., “regulation of T cell proliferation”). Families of similar GO annotations are grouped into boxes; representative GO annotations contained within are indicated next to each box. A zoomed-in view of each network and GO annotation can be viewed in (Additional File 7: Fig. S3). Note that GO biological processes are not the same as pathways, but rather a grouping of molecular activities contributing to a single event

Unexpectedly, cluster 1 was highly enriched in GO biological processes such as “collagen fibril organization” (COL1A1, COL1A2, LOX, COL5A1), “homophilic cell adhesion” (PCDHGA7, PCDH9, PCDH20, CDHR1), and “tissue development” (NGFR, IGFBP5, ELN, TGFB1I1) (Fig. 3a). The majority of protocaderins associated with “homophilic cell adhesion” are involved in neuronal process guidance and adhesion [44]. Of note, a number of processes associated with fibroblast proliferation and migration were also highly enriched (Additional File 6: Table S4).

Transcripts highly expressed in both PEG and Unop groups in cluster 2 were included in GO annotations that involve cell differentiation, Wnt signaling, and cyclic AMP (cAMP) signaling such as “Wnt receptor signaling pathway,” “calcium modulating pathway” (FZD2, WNT11, WNT16), “nervous system development” (RTN4R, GDF11, ARNT2, EDN3), “neural crest cell migration” (SHH, EDN3, SEMA3C, NRTN), and “cAMP metabolic process” (PDE1C, PDE3A, ADCY8) (Fig. 3b).

The transcripts most highly expressed in unoperated control nerves (cluster 3) were primarily enriched in lipid metabolism, myelination, and ion transport functions, such as “fatty acid metabolic process” (FADS3, ACSL1, SCD, LEP), “ion transport” (KCNK5, CLIC5, GRIK3, SLC4A1), and “regulation of action potential” (NFASC, P2RX5, KCNIP1, P2RX3) (Fig. 3c).

As expected from our previous examination of immune responses in nerve allografts [19], the transcripts most highly expressed in cluster 4 were enriched in processes such as “inflammatory response” (GATA3, IL1B, C4A, NFKB1), “regulation of T cell proliferation” (IL2, ZAP70, CD28, IFNG), and “antigen processing and presentation” (RT1-A1, RT1-DB1, B2M, TAP2) (Fig. 3d). Several transcripts in cluster 4 also were found to be associated with muscle tissue development. This was not unexpected since thin layers of tightly adhered muscle and connective tissue surrounding rejected NC PNAs is a common occurrence [7, 17]. Before experimentally excising the PNA, the muscle layer could not be completely trimmed from the PNA without damaging it. Tabular listings of all BiNGO annotations and their associated transcripts can be found in (Additional File 6: Table S4).

### Collagens, cadherins, and metallopeptidases are overrepresented in PEG-fused PNAs

**Fig. 4:**
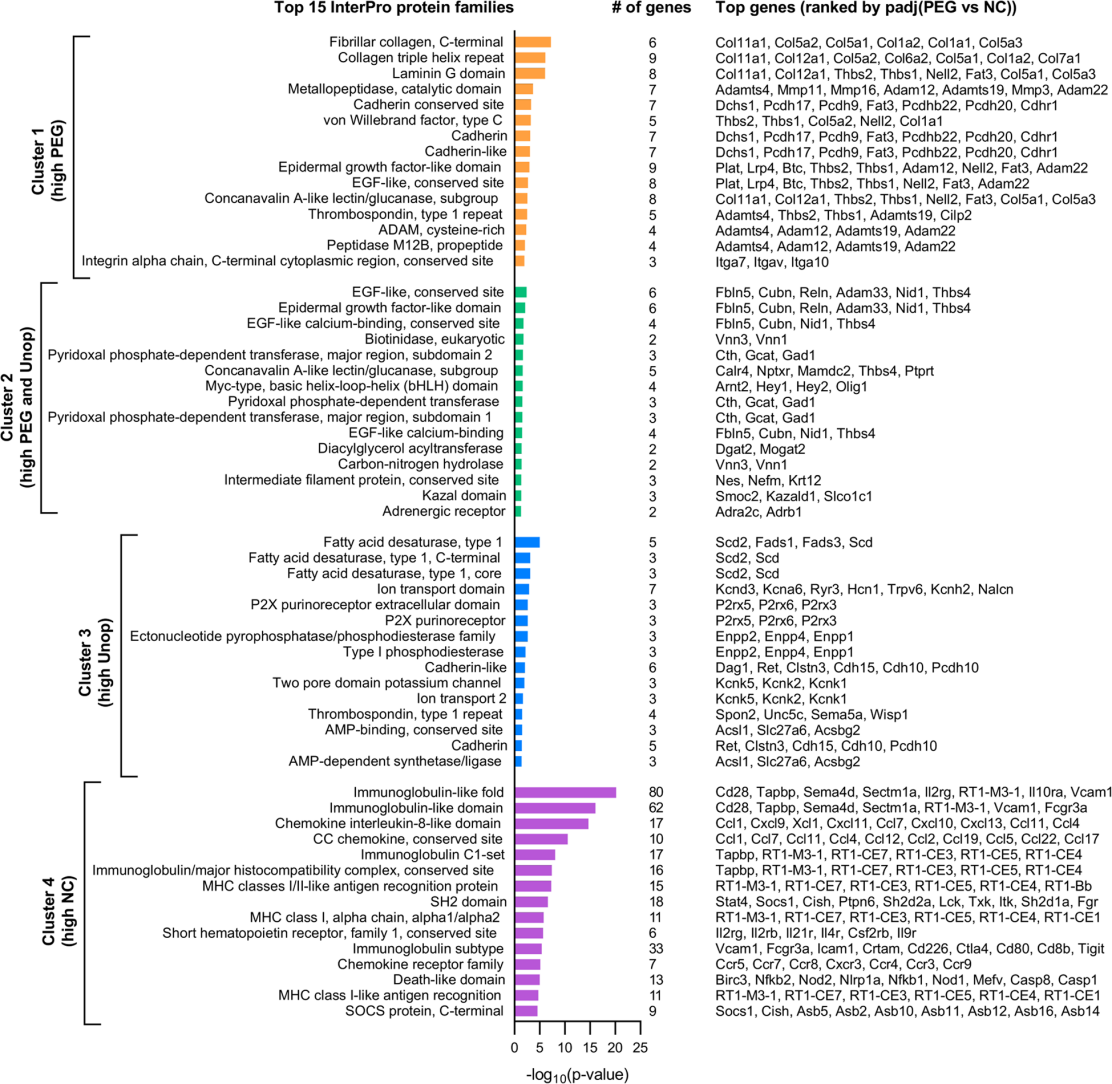
Top 15 overrepresented InterPro protein families in clusters 1–4. InterPro terms in each cluster are ranked by *p* value (Fisher Exact test). The number of transcripts associated with each term as well as the top associated transcripts (ranked by padj (PEG vs. NC comparison)) are indicated. Note that a single transcript may be associated with more than one term

Next, we identified which types of proteins were most highly represented in the list of transcripts that associated with GO annotations. We employed the InterPro database, which classifies proteins by families, domains, and other identifiable features. Upon entering the gene transcript ID’s associated with each GO annotation from each cluster into InterPro via DAVID, we found that the top overrepresented protein families encoded by cluster 1 transcripts included fibrillar collagen, laminin G domains, cadherins, protocadherins, epidermal growth factor (EGF)-like domains, thrombospondin type 1 repeats, metallopeptidases, and integrin alpha chains (Fig. 4). Top cluster 2 proteins included additional EGF-like domains, biotinidases, pyridoxal phosphate-dependent transferases, intermediate filament proteins, and adrenergic receptors, among others. Top cluster 3 proteins were comprised of ion transport domains, fatty acid desaturases, P2X purinoceptors, cadherins, and phosphodiesterases. Lastly, cluster 4 consisted of immunoglobulin-like folds, major histocompatibility class I and II antigen recognition proteins, chemokine interleukin-8-like domains, chemokine receptors, and death-like domains. A full list of all InterPro annotations with associated transcripts can be found in (Additional File 6: Table S4).

Collectively, the overrepresentation of collagens, cadherins, and other proteins involved in extracellular matrix remodeling and cell adhesion is suggestive of enhanced and/or expedited wound healing responses from fibroblasts and/or Schwann cells in PEG-fused PNAs. The results also implied that there may be an important connection between PEG-fusion-mediated changes in extracellular matrix remodeling and attenuated immune responses. It may be possible that PEG-fused PNAs consist of many intact axon/Schwann cell units surrounded by dense accumulation of extracellular matrix that may act as a physical barrier to infiltrating cells. These issues are readdressed in the “Discussion” section.

### Downregulation of the allograft rejection pathway and differential expression of particular integrins in PEG-fused PNAs

**Fig. 5:**
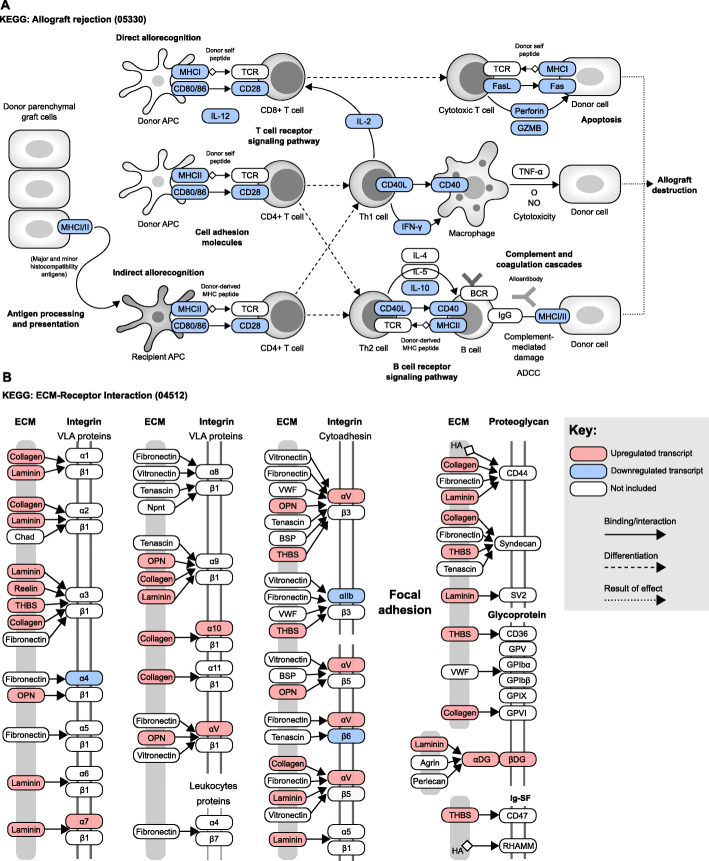
Mapping of RNAseq data (PEG vs. NC) to top KEGG pathways from clusters 1 and 4. **a** Allograft rejection pathway (2nd most highly enriched for downregulated transcripts in the PEG vs. NC comparison) showing key molecules and processes involved in allograft rejection (ordered left to right in the diagram). Upregulated transcripts (pink tiles), downregulated transcripts (blue tiles), and transcripts that are part of the pathway, but not included in our list of DEGs (white tiles) are shown. Solid arrows represent protein interactions, while dashed arrows pointing to or from T cells represent T cell differentiation into effector phenotypes. Dotted arrows point to a subsequent event. **b** ECM-receptor interaction pathway (most highly enriched for upregulated transcripts in the PEG vs. NC comparison) showing binding interactions between extracellular matrix proteins and either integrins, proteoglycans, glycoproteins, or immunoglobulin superfamily (Ig-SF) members. These diagrams have been stylistically modified from the original KEGG diagrams for display purposes

To better understand the relationship between the transcripts that are differentially expressed in the PEG vs. NC comparison, we used DAVID to identify the most highly enriched KEGG pathways associated with each cluster (Additional File 6: Table S4). We then selected the most enriched KEGG pathway in cluster 1 (“ECM-receptor interaction”) and the second-most enriched KEGG pathway for cluster 4 (“Allograft rejection”), and mapped all 2180 DEGs from the PEG vs. NC comparison into two pathway diagrams (Fig. 5a, b). The diagram for the most enriched KEGG pathway in cluster 4 (“cytokine-cytokine receptor interaction”) was too large to include as a figure.

We found that all DEGs that mapped to the “Allograft rejection” pathway were downregulated (Fig. 5a). These transcripts were involved in functions such as antigen presentation in both direct and indirect allorecognition pathways, T cell and B cell receptor signaling, and donor cell killing. In the “ECM-receptor interaction” pathway, all of the mapped DEGs were upregulated with the exception of those that encoded alpha 4, alpha IIb, and beta 6 integrin subunits (Fig. 5b). These integrins are integral to T cell infiltration across the endothelium, platelet aggregation during wound healing, and fibronectin binding, respectively [45]. Collagens, laminins, reelins, thrombospondins, and SPP1 were highly prominent among upregulated DEGs, as were alpha 7 and alpha V integrin subunits, which encode proteins that form interactions with basement membranes and a wide variety of other ligands.

These results implied that the majority of the pathways necessary for allorejection were significantly impaired in PEG-fused PNAs. They also provided further support for the interpretation that PEG-fusion-mediated changes in the extracellular matrix might contribute to an immunosuppressive environment in PEG-fused PNAs.

### Factors commonly involved in immunosuppression are upregulated in PEG-fused PNAs

From functional annotation analyses, we identified several DEGs from cluster 1 that could contribute to an immunosuppressive environment in PEG-fused PNAs, including CD24, thrombospondin 1 (THBS1), thrombospondin 2 (THBS2), and CD276.

CD24 is a glycophosphatidylinositol (GPI)-linked protein presented on the surface of a variety of cells associated with the nervous and immune system that contribute to a range of functions [46]. In the immune system, CD24 expressed in antigen-presenting cells binds to the sialic acid binding lectin Siglec-10 on macrophages [47]. Siglec-10 closely associates with the tyrosine phosphatases SHP-1 and SHP-2, which negatively regulate nuclear factor kappa B (NF-κB) signaling. Thus, interaction of Siglec-10 with CD24 results in suppression of TLR-mediated inflammatory signaling in response to tissue damage, as well as in phagocytic clearance. The immunosuppressive microenvironment of many tumors correlate with CD24 overexpression. CD24 overexpression in PEG-fused allografts may contribute to similar effects.

THBS1 and THBS2 are large homotrimeric extracellular matrix binding proteins transiently expressed in fibroblasts, Schwann cells, and endothelial cells. They support remodeling and assembly of the collagen matrix (among many other functions) following tissue damage and inflammation [48, 49]. Both proteins are structurally similar, and their overexpression confers potent anti-inflammatory properties [50, 51]. These include drastically reduced inflammation, T cell infiltration, production of IFN-γ, and differentiation of T cells into their effector phenotypes. The latter mechanism likely occurs through interaction with the CD47 antigen on the T cell surface. Binding of CD47 may interfere with antigen-mediated signaling in T cells and sensitize them to Fas-mediated apoptosis.

The type I transmembrane protein CD276/B7-H3 is an influential immune checkpoint that is a component of the B7/CD28 co-stimulatory activation pathway in T cells [52]. CD276 is commonly expressed in fibroblasts and endothelial cells following induction by antigen-presenting cells. In T cells, a combination of B7-1/2 and CD28 co-stimulation with the peptide/MHC complex is required for full activation of Th1 responses [10]. CD276 can bind to CD28 on T cell surfaces to inhibit T cell activation of Th1 responses and proliferation by serving as a co-inhibitory molecule to suppress the transcriptional activities of nuclear factor kappa B (NF-kB), nuclear factor of activated T cells (NFAT), and AP-1. This suppression prolongs allograft survival [53]. Overexpression of CD276 is a common mechanism by which tumors evade the adaptive immune response in numerous types of cancer [52].

**Fig. 6:**
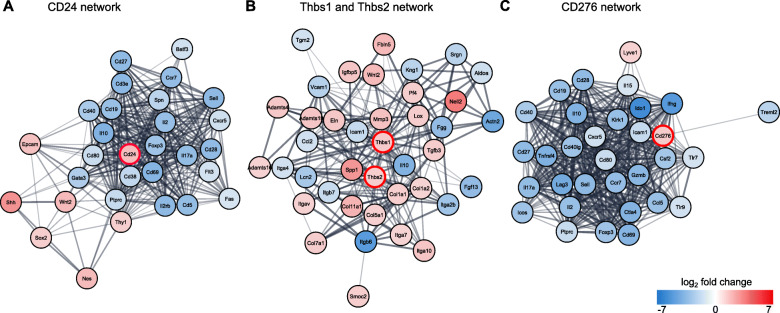
STRING protein-protein interaction networks of immunosuppressive DEGs. STRING protein-protein interaction networks of upregulated (red) and downregulated (blue) DEGs directly associated with a CD24, **b** THBS1 and THBS2, and **c** CD276. The list of transcripts used to create these networks was derived from all DEGs associated with clusters 1, 2, and 4. Each node indicates a specific protein, shaded according to its log_2_ fold change value (PEG vs. NC comparison). Pink = upregulated, blue = downregulated. Distances between nodes indicates greater or lesser association between proteins, while the thickness of the lines between nodes indicates the confidence score of the interaction (thicker = greater confidence, thinner = lesser confidence). The positions of CD24, THBS1, THBS2, and CD276 in each network are indicated by red borders

Next, we determined which other proteins among the DEGs within the PEG vs. NC comparison associate with those encoded by CD24, THBS1, THBS2, and/or CD276 by creating STRING protein-protein interaction networks (Fig. 6a–c) [29]. STRING displays networks that have known or predicted protein-protein interactions, based on experimental determination, computational prediction, and public text-mining. All transcripts associated with clusters 1, 2, and 4 served as input to generate the networks; their fold change values indicate up- or downregulation of each transcript. As shown in Fig. 6a, CD24 associates with Sonic Hedgehog (SHH) and SOX2*.* SOX2, is a transcription factor that, in part, promotes the demyelinating Schwann cell state [5]. SOX2 also directly upregulates CD24 expression by binding to its promoter [54]. CD24 associates with a number of cluster 4 downregulated transcripts that are linked to immune responses, including CD28, IL2, and CD3E. THBS1 and THBS2 were primarily associated with upregulated extracellular matrix transcripts that include collagens, integrins, and metalloproteases (Fig. 6b). Downregulated transcripts that associated with THBS1 and THBS2 are involved in coagulation and adhesion to endothelial cells such as intercellular adhesion molecular 1 (ICAM1) and vascular adhesion molecule 1 (vcam1) [10]. CD276 associates with a wide variety of downregulated immune response transcripts from cluster 4, which include chemokines, cytokines, co-stimulatory molecules, and adhesion molecules (Fig. 6c).

Following traumatic nerve injury, fibroblasts proliferate and upregulate fibrillar collagen production to excessive amounts as a component of scar formation [21, 55]. Excessive collagen production can act as a mechanical barrier to axonal regeneration following injury. A dense collagen matrix also can physically block T cells from infiltrating certain types of solid tumors [56]. T cells preferentially accumulate in areas of low collagen density and have greater difficulty migrating and contacting target cells in areas of high collagen density. A recent study has shown that dense collagen also inhibits the ability of T cells to proliferate, to produce cytotoxic molecules and to kill tumor cells [57]. Adhesion of cells to collagen matrices via integrins is also stimulated by THBS1 and THBS2 [48, 49]. CD276 expressed on cells within the PEG-fused PNA microenvironment may negatively regulate T cell activation [53].

**Fig. 7:**
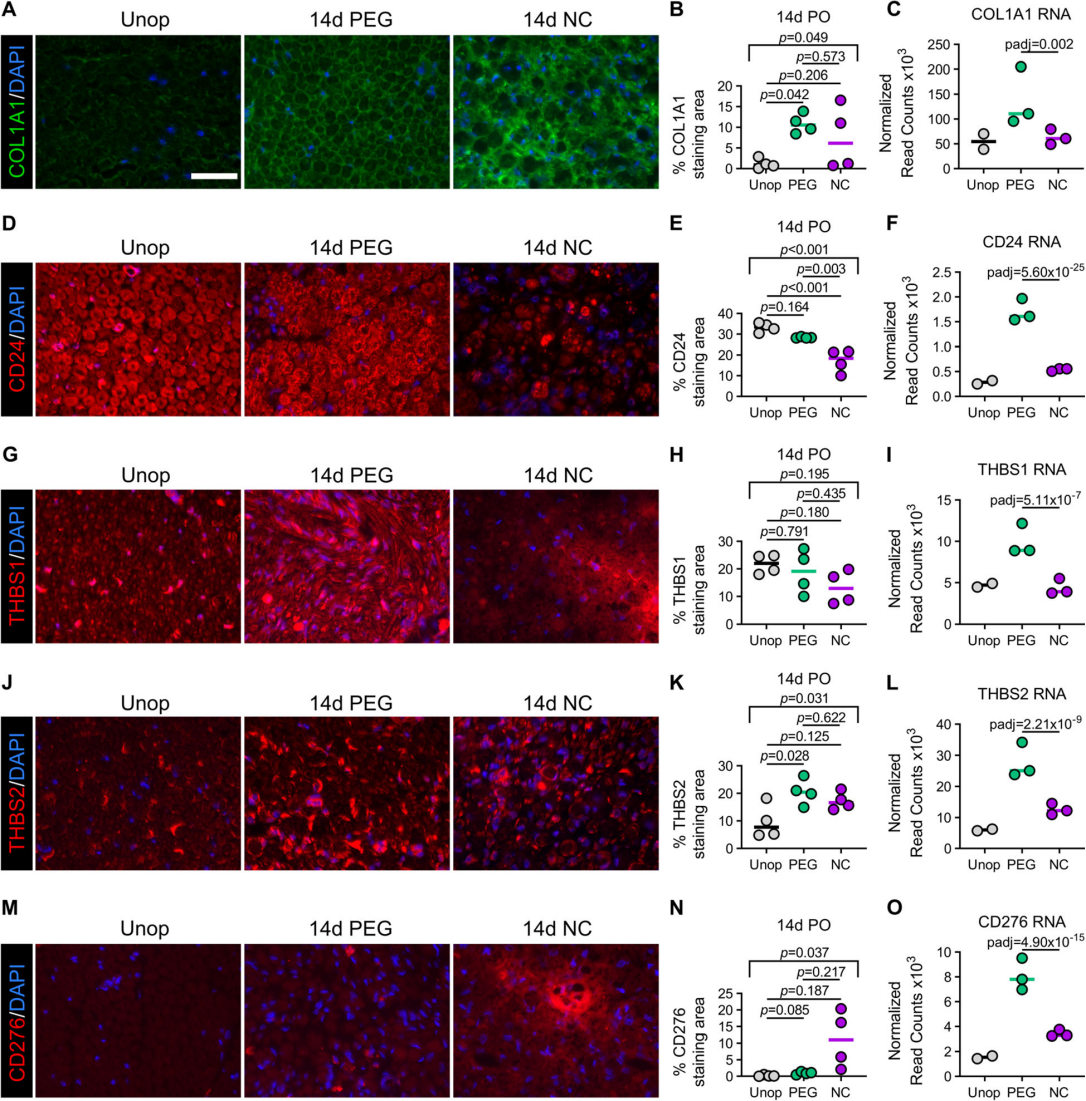
Immunohistochemical analyses of cell adhesion and extracellular matrix DEGs. **a** Fluorescence images showing cross-sections of unoperated control nerve, 14 days PO PEG-fused PNA, and 14 days PO NC PNA immunostained for COL1A1 (green). **b** Percent (%) COL1A1 staining area comparisons at 14 days PO (*n =* 4 per treatment group). **c** Normalized read counts of COL1A1 RNA transcripts at 14 days PO. Fluorescence images showing immunostaining for CD24 (red) in cross-sections. **d** Fluorescence images showing immunostaining for CD24 (red). **e** Percent (%) CD24 staining area comparisons at 14 days PO (*n =* 4 per treatment group). **f** Normalized read counts of CD24 RNA transcripts at 14 days PO. Fluorescence images showing immunostaining for CD24 (red) in cross-sections. **g** Fluorescence images showing immunostaining for THBS1 (red). **h** Percent (%) THBS1 staining area comparisons at 14 days PO (*n =* 4 per treatment group). **i** Normalized read counts of THBS1 RNA transcripts at 14 days PO. **j** Fluorescence images showing immunostaining for THBS2 (red). **k** Percent (%) THBS2 staining area comparisons at 14 days PO (*n =* 4 per treatment group). **l** Normalized read counts of THBS2 RNA transcripts at 14 days PO. **m** Fluorescence images showing immunostaining for CD276 (red). **n** Percent (%) CD276 staining area comparisons at 14 days PO (*n =* 4 per treatment group). **o** Normalized read counts of CD276 RNA transcripts at 14 days PO. Nuclei are stained with DAPI (blue). Scale = 50 μm. In graphs, data are shown as mean (horizontal bar) with each individual data point per animal. In **b**, **e**, **h**, **k**, and **n**, overall one-way ANOVA *p* values are shown above brackets at each time point, with multiple comparisons *p* values between treatment groups displayed underneath

These results prompted a immunohistochemical analysis to validate and compare the protein levels of collagen type I alpha type 1 (COL1A1), which is commonly found in peripheral nerves, as well as those of CD24, THBS1, and THBS2. We also examined protein levels of CD276. As shown in Fig. 7a and b, COL1A1 was significantly and consistently increased in PEG-fused PNAs relative to unoperated control nerves in regions where Schwann cell-axon units are typically located. In NC PNAs, COL1A1 levels were highly variable, with some animals showing high levels (i.e., PEG-fused PNAs) and others showing relatively low levels (i.e., similar to those in unoperated controls). CD24 was very strongly expressed at the protein level in both unoperated control nerves and PEG-fused PNAs (Fig. 7d, e)—values significantly higher than observed in NC PNAs (one-way ANOVA *F*(2,9) = 23.25, *p* < 0.001, Tukey’s multiple comparisons test *p =* 0.003). In contrast, CD24 RNA expression in unoperated control nerves was relatively low compared to that in PEG-fused PNAs (Fig. 7f). THBS1 RNA expression was significantly higher in PEG-fused PNAs compared to unoperated control nerves and negative control PNAs. However, THBS1 was strongly expressed at the protein level in all treatment groups (Fig. 7g–i). Although THBS2 levels were significantly higher in PEG-fused PNAs compared to unoperated control nerves (one-way ANOVA *F*(2,9) = 5.24, *p* = 0.031, Tukey’s multiple comparisons test *p =* 0.028), there was no significant difference between PEG-fused PNAs and NC PNAs (Fig. 7j, k). Despite the fact that CD276 RNA expression is increased in PEG-fused PNAs compared to negative control PNAs, protein expression of CD276 was very low in PEG-fused PNAs and highly variable in negative control PNAs (Fig. 7m–o).

These results suggested that, with the exception of CD276, each of the extracellular matrix and cell adhesion proteins examined via immunohistochemistry were elevated in PEG-fused PNAs. CD24 in particular showed the greatest difference in expression between PEG-fused PNAs and negative control PNAs. Contrasts between RNA expression and protein expression in either treatment group may be due to post-transcriptional regulatory effects.

## Discussion

### Summary of findings

We previously reported that PEG-fused sciatic PNAs in rats maintained morphological and electrophysiological continuity, did not undergo Wallerian degeneration, maintained large-caliber myelinated axons that were not rejected, maintained highly innervated distal neuromuscular junctions, and had improved behavioral recovery for up to 42 days PO [17]. We also reported that PEG-fused PNAs had reduced innate and adaptive inflammatory responses in the form of significantly reduced T cell and macrophage infiltration, MHC I and II expression, expression of IFN-γ and CXCL11, and consistently reduced apoptosis [19].

In the present study, we assess PEG-fused PNAs using transcriptomic analyses. Differential gene expression, GO biological processes, KEGG pathways, and InterPro protein families are compared between PEG-fused PNAs and NC PNAs at 14 days PO—a time when inflammatory and rejection responses were easily detectable in NC PNAs. These data are then compared with data collected from unoperated control sciatic nerves.

Transcriptomic analyses in PEG-fused PNAs compared to NC PNAs reveal downregulation of numerous T cell and macrophage-associated transcripts and upregulation of many Schwann cell-associated transcripts in both myelinating and demyelinated states (Fig. 1a–h). Compared to unoperated control nerves, we observe upregulation in PEG-fused PNAs of the same T cell, macrophage, and demyelinated Schwann cell-associated transcripts and downregulation of myelinating Schwann cell-associated transcripts (Fig. 1a, b; Additional File 4: Table S2). GO analyses reveal upregulated collagens, cadherins, and metallopeptidases (i.e., extracellular matrix remodeling, cell adhesion and tissue development) (Fig. 3a). All transcripts commonly involved in allograft rejection pathways are downregulated, whereas several transcripts commonly involved in immunosuppression or immune evasion are highly upregulated in PEG-fused PNAs compared to NC PNAs and/or unoperated control nerves (Figs. 5a, b; 6a–c; and 7a–f). These transcripts include CD24, THBS1, THBS2, and CD276. All except CD276 are upregulated at the protein level in PEG-fused PNAs, although only CD24 is significantly upregulated (one-way ANOVA *F*(2,9) = 23.25, *p* < 0.001, Tukey’s multiple comparisons test *p =* 0.003) relative in PEG-fused PNAs relative to negative control PNAs. Some transcripts involved in extracellular matrix remodeling or cell adhesion are also upregulated, suggesting that these processes may have a role in PEG-mediated immunosuppression.

### Modulation of T cell activation and allorejection responses

Allogeneic tissue rejection is due to complex interactions among antigens, MHC molecules, adhesion molecules, cytokines, chemokines, and transcription factors. Inhibiting any one of these interactions often leads to immunosuppressive effects and prolonged allograft survival [10, 58]. T cell effector responses against donor cells (T cell proliferation, cytokine production, apoptosis, etc.) require forming immunological synapses with peptide/MHC and T cell receptor (TCR) complexes with cytokines and co-stimulatory molecules such as CD28, CD80 (B7.1), CD86 (B7.2), CD40, and CD40LG [10]. CD28 expressed on T cells binds to CD80 or CD86 on donor or host antigen presenting cells to promote signaling cascades that control T cell proliferation, differentiation into effector types, and cytokine production [10]. CD40LG, when expressed on T cells, binds to CD40 on other host T cells or antigen presenting cells such as macrophages and B cells to stimulate their differentiation and activation of effector responses. The CD28 co-stimulation pathway can be inhibited by the homologous receptor CTLA4, which competes with CD28 for binding of CD80 or CD86 [58]. Co-stimulatory blockades using CTLA4-immunoglobulin fusions or anti-CD40LG antibodies can reduce rejection-associated immune responses [58]. Interactions between the ligand CD274 and the receptor PDCD1 on cytotoxic T cells blocks their activation—a mechanism to evade immune rejection often used by tumors [58].

We report that each of these co-stimulatory molecules and their ligands (including CTLA4, PDCD1 and CD274) are significantly downregulated in PEG-fused PNAs compared to NC PNAs (Fig. 1a). This suggests that they are unlikely to play an important role in T cell suppression of PEG-fused PNAs. CD276 RNA is highly upregulated in PEG-fused PNAs and is involved in co-inhibition of the CD28 pathway (Additional File 5: Table S3) [52]. CD276 expression is inhibited by a microRNA, miR-29, that also inhibits extracellular matrix production and cell proliferation [48, 59]. These data suggest a linkage between fibrogenesis and modulation of the immune response. However, protein expression of CD276 is lower in PEG-fused PNAs relative to negative control PNAs. In addition to differences in post-translational regulatory effects, these results may be due to a reduced presence of antigen presenting cells in PEG-fused PNAs.

T cell proliferation, survival, and effector functions are largely controlled by cytokine stimulation, especially IL-2, IL-12, IFN-γ, and IL-17 [10]. Their associated signaling pathways are regulated via JAK/STAT signaling and the transcription factors NFAT and NF-kB, which are both downregulated in PEG-fused PNAs. IL-2 is produced primarily in activated Th1 cells and functions as both an autocrine and a paracrine factor by stimulating rapid proliferation and differentiation of both CD8 and CD4 effector T cells [32]. IL-12 is produced by antigen-presenting cells such as macrophages and dendritic cells and acts as a powerful inducer of IFN-γ in Th1 and cytotoxic T cells [31]. IFN-γ serves many functions in rejection, such as maintaining T cell survival, inducing MHC expression in nearby cells and inducing macrophages to produce pro-inflammatory cytokines and ROS [33]. IL-17 is produced by Th17 cells and stimulates proinflammatory cytokine production, antigen presentation, and it may suppress myelin production in Schwann cells as well [60]. We observed that PEG-fused PNAs exhibit significant downregulation of each of these critical cytokines, as well as effector molecules FASL, PRF1, and GZMB that are commonly employed by cytotoxic T cells to kill donor cells (Fig. 1a) [8, 10].

The production of these cytokines in T cells can be inhibited via IL-10 and/or TGF-β1 stimulation by regulatory T cells (Tregs). Treg-mediated suppression of Th1 and Th2 responses is a common method of immune evasion in both tolerated allografts as well as in many cancers [34]. However, we observe that transcription of IL-10 and TGF-β1, as well as FOXP3, the transcription factor driving Treg differentiation [34], are each significantly downregulated in PEG-fused PNAs (Fig. 1a). These results suggest that inhibition of innate inflammatory processes that precede T cell recruitment, suppression of antigen presentation, and/or physical blockades of immune cell migration are responsible for PEG-fusion-mediated immunosuppression in PNAs.

When comparing PEG-fused PNAs and unoperated control nerves, we found that each of the factors described above are upregulated in PEG-fused PNAs (Additional File 4: Table S2). These results indicate that an adaptive immune response is still present within PEG-fused PNAs, although at significantly lower magnitude than in NC PNAs.

### Regulation of damage-recognition responses in innate immunity

An innate host inflammatory response to surgical injury typically proceeds other adaptive and innate immune responses to donor allogeneic tissues [8, 9]. Following injury and during Wallerian degeneration, damaged and/or degenerating cells and axons within donor PNAs release damage-associated molecular patterns (DAMPs) such as HMGB1 and ATP [3]. These substances are recognized by TLRs expressed by resident innate immune cells (such as macrophages) or non-immune cells (such as Schwann cells) [3]. DAMP binding to these receptors triggers innate immune cells and activated Schwann cells to release an inflammatory milieu via signaling pathways such as NF-kB [3]. This, in turn, recruits and potentiates adaptive immune cells to carry out a rejection response. In Schwann cells, these events coincide with differentiation from a myelinating state to a demyelinated state that facilitates myelin clearance and regeneration of axonal sprouts [3, 5].

We observe that some toll-like receptors (including TLR1, TLR4, and TLR9) are downregulated in PEG-fused PNAs when compared to NC PNAs (Fig. 1a; Additional File 4: Table S2). We observe downregulation of mediators of innate inflammatory responses (e.g., macrophage differentiation), including the transcription factor, NFKB1, and its target inflammatory cytokine IL1B. NOS2, which contributes to oxidative damage [8], is also downregulated. These results confirm and expand our previous immunohistochemistry-based assessment of reduced innate inflammatory responses in PEG-fused PNAs [19]. That is, IL1B and NOS2 are commonly produced by the classically activated M1 macrophage phenotype [36]. Their reduction, when combined with the observed increased expression of Arginase I and MRC1 in PEG-fused PNAs, suggests a shift in macrophage polarization from a pro-inflammatory M1 state to an alternatively activated anti-inflammatory M2 state. This shift is critical as the M2 state is a major contributor to wound healing [36]. Numerous axons and Schwann cells in the PNA remained intact, functional, and nondegenerate following PEG-fusion repair [17, 19]. Thus, an effective response to tissue damage by resident Schwann cells or macrophages is impaired—possibly resulting from the significant prevention of tissue damage through PEG-fusion. Other transplanted tissues treated with PEG via organ storage solution or intraluminal infusion have reduced inflammation and improved cell viability [61]. Therefore, diminished ischemia reperfusion injury as a result of reduced mitochondrial swelling and ROS production, as well as restoration of membrane integrity in these tissues, may also contribute to the amelioration of DAMP-mediated responses in PEG-fused PNAs. The upregulation of M1-associated inflammatory factors in PEG-fused PNAs compared to unoperated control nerves suggests that damage-recognition and innate immune responses in PEG-fused PNAs are attenuated, rather than eliminated.

Our analyses of Schwann cell-associated transcripts reveal upregulation of factors that drive both the myelinating Schwann cell state (e.g., SOX10, MBP, CDH2) as well as the demyelinating state (e.g., JUN, SOX2, NGFR) within PEG-fused PNAs compared to NC PNAs (Fig. 1b). This elevation in common Schwann cell-associated transcripts may underlie the longer survival of Schwann cells in PEG-fused PNAs. We also observed enrichment of GO processes that involve both positive and negative regulation of tissue development and cell differentiation (Fig. 3a, b). This is supported by our previous electron microscopic observation [17] that showed within cross-sections of PEG-fused PNAs sampled at 21–42 days PO, there exists regions of successfully PEG-fused axons with myelinating Schwann cells as well as regions unsuccessfully PEG-fused that have undergone Wallerian degeneration. Compared to unoperated control nerves, the downregulation of myelinating Schwann cell-associated genes likely contributes to unsuccessfully PEG-fused regions. Generally, PNAs that have greater PEG-fusion success and greater functional recovery at 42 days PO have a greater ratio of large-caliber myelinated axons to small-caliber demyelinated axons [17]. A large population of myelinating Schwann cells and resident macrophages in successfully PEG-fused PNAs by 14 days PO are not likely to be associated with axons that have undergone Wallerian degeneration. These data suggest that these Schwann cells and nearby resident macrophages do not activate an inflammatory signaling cascade because they lack interaction with DAMPs that would have been otherwise released from degenerating axons.

In the nervous system, CD24 is known to be expressed in Schwann cells and developing neurons [46]. CD24 inhibits the outgrowth of dorsal root ganglion neurites and adhesion via its interaction with sialic acid-binding lectin L1 on axonal membranes [62]. At the mRNA level, high expression of CD24 in PEG-fused PNAs relative to unoperated control nerves may be related to the processes of neurite outgrowth regulation that follows degeneration of unsuccessfully PEG-fused axons. On the other hand, high protein levels of CD24 in unoperated control nerves and PEG-fused PNAs might be more closely associated with maintaining adhesion of axons with myelinating Schwann cells. The significant decrease in CD24 in NC PNAs that have undergone Wallerian degeneration supports this interpretation. The slight increase of CD24 transcript expression in NC PNAs relative to unoperated control nerves suggests that CD24 may be also associated with either Schwann cell or immune cell activation of demyelinated states and/or Wallerian degeneration. CD24, when bound to Siglec-10 on immune cells, inhibits the immune response to common cytoplasmic and nuclear DAMPs [46, 47]. However, this effect seems to be at odds with the proinflammatory response to DAMPs typically associated with demyelinated Schwann cells following injury.

### Factors controlling chemotaxis and extravasation into allograft tissue

Following the initial inflammatory response, immune cells migrate via chemotaxis to extravasate through the endothelial lumen to reach the graft tissue [10]. These processes are largely governed by chemokines such as CCL1/2/5 and CXCL9/10/11, as well as intercellular adhesion molecules such as selectins, ICAMs, and VCAMs that mediate cell rolling and diapedesis [10]. After passing through the lumen, the extracellular matrix surrounding the entry site is degraded by metallopeptidases such as MMP3 to grant full entry. We report that each major chemokine that attracts T cells and macrophages, as well as most adhesion molecules involved in extravasation, are downregulated in PEG-fused PNAs compared to NC PNAs (Fig. 1a; Additional File 4: Table S2). These results suggest that immune cells may not easily penetrate the inner region of a PEG-fused PNA.

Unexpectedly, a number of metallopeptidases (e.g., MMP3, 11 and 23) are upregulated in PEG-fused PNAs (Fig. 4; Additional File 4: Table S2). These molecules typically are expressed during injury and inflammation [63]. These data suggest that metallopeptidase overexpression in PEG-fused PNAs plays an important role in extracellular matrix remodeling during resolution of injury and wound healing.

One of the most prominent findings of this study is the substantial upregulation of many extracellular matrix components in PEG-fused PNAs compared to NC PNAs and unoperated control nerves. These include an extensive array of collagens and fibroblast growth factors (Figs. 3a and 4; Additional File 6: Table S4). The extracellular matrix not only provides structural support to tissue components but also participates in a wide variety of signaling events that regulate the behaviors of nearby cells. Collagens, laminins, and fibronectins make up the main components of peripheral nerve ECM [55]. The epineurium and perineurium of peripheral nerves is composed mostly of fibrillar type I, II, and III collagens synthesized by fibroblasts. The endoneurium contains the Schwann cell basement membrane, consisting of type IV and V collagens as well as laminins that assist in controlling myelination [55, 64]. Several other types of collagens, such as type XI, serve as networks that link additional fibrillar collagen strands or promote their polymerization [65].

Our results show that type I collagen is highly upregulated at both transcriptional and protein levels in PEG-fused PNAs (Figs. 4 and 7a–c). We suggest that a large number of myelinating Schwann cells in PEG-fused PNAs do not respond to injury stimuli, but fibroblasts in PEG-fused PNAs do respond. Excessive collagen production by these fibroblasts might create an environment in which intact Schwann cell-axon units are surrounded by a dense collagen matrix. This would physically block T cells from accessing Schwann cells and fibroblasts, as well as inhibit their molecular activities. High expression of THBS1 and THBS2, which are integral to collagen formation processes and can have potent T cell suppression properties, might further bolster protection [50, 51]. One might then assume that excessive collagen production in rejected PNAs not treated with PEG would also provide the same immunomodulatory effect. In contrast, we suggest that donor fibroblasts in untreated PNAs are also targets for rejection, thereby limiting collagen production and/or decreasing the density of collagen deposition. A combination of Schwann cell quiescence and other immunosuppressive properties conferred by PEG treatment may inhibit inflammatory signaling.

### Antigen processing and presentation in PEG-fused PNAs

The coordination of antigen processing and presentation of MHC molecules in donor cells and host antigen presenting cells is carried out by a number of proteases, transport molecules, and binding proteins [10]. Endogenous peptide fragments are generated in cytoplasmic proteasomes and are then transported into the endoplasmic reticulum via TAP1 and TAP2 proteins, where they are loaded onto MHC class I molecules [10]. Exogenous peptide fragments enter the cell via endocytosis, where they are cleaved within lysosomes by proteases such as cathepsins D and L before being loaded onto MHC class II molecules. The expression of MHC class I and II subunits is controlled by the transcription factors NOD-like receptor C5 (NLRC5) and class II major histocompatibility complex transactivator (CIITA), respectively. Each of these are upregulated by IFN-γ stimulation during injury and inflammation [66, 67].

Our transcriptomic analyses expand upon our previous IHC analyses [19] to show that PEG-fused PNAs have significantly reduced MHC I and II protein expression compared to NC PNAs. We further report that the transcription of a number of integral components of antigen processing and presentation are also significantly downregulated. These include TAP1, TAP2, NLRC5, CIITA, and a wide variety of MHC subunits such as beta-2-microglobulin (B2M), RT1-DB1, RT1-CE3, and RT1-M3-1 (Fig. 4; Additional File 4: Table S2). These results suggest that the ability of donor cells to present antigen to host T cells is severely compromised, thereby decreasing their immunogenicity.

Cathepsins D and L are upregulated in PEG-fused PNAs (Additional File 4: Table S2). Aside from their assistance in antigen processing, these proteases perform a number of additional functions within tissues [68, 69]. Cathepsins D and L are typically stored in cytoplasmic lysosomes. But each can be secreted into the extracellular environment, where they cleave matrix components such as fibronectins, collagens, and laminins [68, 69]. Procathepsin D has been shown to stimulate proliferation and motility in stromal fibroblasts [70]. Overexpression of cathepsin D also has been associated with impaired antigen processing of T cell epitopes generated from myoglobin [71]. Dendritic cells lacking cathepsin D show enhanced presentation of these epitopes [71]. We suggest that increasing the concentration of cathepsin D in PEG-fused PNAs may lead to an increased number of cleavage sites on peptides, resulting in their destruction rather than their appropriate cleavage into presentable fragments.

Our results suggest that both PEG-fused PNAs and unoperated control nerves significantly upregulate adenylyl cyclase 8 (ADCY8) and phosphodiesterase 1C (PDE1C) that together catalyze cyclic AMP (cAMP) production and turnover (Additional File 4: Table S2) [72]. The myelinating state of Schwann cells is largely controlled by axonal contact through interactions between laminins on the Schwann cell basement membrane and the G-protein coupled receptor GPR126 [73]. This interaction stimulates cAMP in Schwann cells to stimulate transcription of promyelinating genes, such as early growth response 2 (EGR2) [74]. cAMP also activates protein kinase A (PKA), which inhibits induction of MHCII by phosphorylating its central regulatory transcription factor, CIITA [75]. Myelinating Schwann cells do not express MHCII but rather express MHCI at low levels [76, 77]. In contrast, demyelinated Schwann cells express high levels of MHCII and upregulate expression of MHCI. PEG-fusion may affect the myelinating state of Schwann cells via cAMP signaling. All these data suggest that a large number of Schwann cells in PEG-fused PNAs may have reduced immunogenicity solely due to their myelinating state.

## Conclusions

This study is the first to examine at the molecular level why PEG-fused PNAs might be immunotolerant even though they are neither immunosuppressed nor tissue-matched. This study is also the first to identify a possible role for extracellular matrix remodeling in the immunosuppression of PEG-fused PNAs. Our data show that PEG-fused PNAs upregulate extracellular matrix remodeling, cell adhesion, tissue development processes, as well as Schwann cell-associated transcripts in both myelinating and demyelinated states, while significantly downregulating innate and adaptive immune responses underlying allograft rejection. We suggest that maintenance of myelinating Schwann cell-axon units, in combination with extracellular matrix component production and reduced responses to cellular injury, contribute to an immunosuppressive environment within PEG-fused PNAs. The knowledge gained by these initial analyses of differential gene expression should generate new hypotheses aimed at understanding molecular mechanisms that underlie the immune tolerance of PEG-fused PNAs. Whether PEG-fused PNAs maintain their immunosuppressive effects in MHC-mismatched donor-host combinations is a subject of ongoing studies. Future transcriptomic studies of PEG-fused PNAs would benefit from single-cell sequencing and flow cytometry analyses to determine cell-specific expression of the transcripts and/or proteins identified from this current whole-tissue examination.

After ablation-type PNIs, recovery of lost behaviors is often poor and clinical outcomes have not significantly improved for decades despite advances in biomedical technologies [2, 7]. PEG-fusion of PNAs produces dramatic recovery within weeks of lost behaviors because allografts are not rejected in the absence of tissue matching and/or immune suppression. We suggest that PEG-fused PNAs have substantial potential to produce a paradigm shift in the clinical treatment of ablation-type PNIs.

## Supplementary information

**Additional file 1: Fig. S1.** (FigS1_Exp_Design.pdf). Outline of the experimental design of the study. PEG-fused sciatic nerve allografts (“PEG”, *n*=3 animals), negative control sciatic nerve allografts (“NC”, *n*=3 animals) from outbred Sprague Dawley rats were excised and sampled 14d post-operatively. Both treatment groups were compared to Unoperated Control sciatic nerves (“Unop”, *n*=2 animals). Total RNA was extracted from each sample via homogenization with TRIzol (Ambion) and a RNeasy Mini kit (Qiagen). Libraries were prepared using a TruSeq Stranded mRNA kit (Illumina), which were then sequenced on a HiSeq 3000 unit (Illumina). Analysis of read results was carried out sequentially by FastQC, Tophat2, HTSeq-count, and DESeq2 software. Subsequent analyses were performed via DAVID and the STRING application for Cytoscape software. Validation of candidate genes was performed via RT-qPCR, using the same treatment groups.

**Additional file 2: Table S1.** (RNAseq_Reads_Table.xlsx). Read mapping and alignment results for all samples used for RNAseq. The ID of each animal is indicated underneath the sample name in parentheses.

**Additional file 3: Fig. S2.** (FigS2_Count_Analyses.pdf). Distribution and correlation of normalized read counts among samples. (A) Violin plots showing the distribution of all log_2_ normalized read counts for each gene transcript in each sample. The median is indicated by a solid horizontal line, and the upper quartile is indicated by a dashed horizontal line. The plots show that the majority of genes in each sample share similar expression profiles and are not differentially expressed. (B) Principle component analysis (PCA) allowing clustering of samples based on variation in expression profiles of all protein coding genes. Samples with similar expression profiles cluster together more closely. (C) Sample-to-sample correlation matrix comparing the gene expression profiles of all coding genes between all individual samples, using normalized read counts. White indicates a Pearson correlation coefficient of <0.6 (lower correlation); black indicates a coefficient of 1 (high correlation).

**Additional file 4: Table S2.** (Differential_Expression_Results.xlsx). Excel tables showing (Tab S1) all normalized read counts for all samples and associated gene transcripts; (Tab S2) PEG vs NC differential expression results; (Tab S3) PEG vs Unop differential expression results; (Tab S4) NC vs Unop differential expression results.

**Additional file 5: Table S3.** (DEG_Heatmap_Clusters.xlsx). Excel tables showing: (Tab S1) Z-scores of all 2,180 transcripts extracted from the DEG heatmap displayed in Fig. 2A, which heatmap cluster each transcript is associated with (Cluster 1, 2, 3, or 4) and the PEG vs NC differential expression results for each transcript; (Tab S2) all transcripts associated with Cluster 1 and their PEG vs NC differential expression results; (Tab S3) all transcripts associated with Cluster 2 and their PEG vs NC differential expression results; (Tab S4) all transcripts associated with Cluster 3 and their PEG vs NC differential expression results; (Tab S5) all transcripts associated with Cluster 4 and their PEG vs NC differential expression results.

**Additional file 6: Table S4.** (GO_KEGG_InterPro_Clusters.xlsx). Excel tables showing (Tabs S1-S3) GO, KEGG and InterPro results for Cluster 1 (high expression in PEG); (Tabs S4-S6) GO, KEGG and InterPro results for Cluster 2 (high expression in PEG and Unop); (Tabs S7-S9) GO, KEGG and InterPro results for Cluster 3 (high expression in Unop); (Tabs S10-S12) GO, KEGG and InterPro results for Cluster 4 (high expression in NC).

**Additional file 7: Fig. S3.** (FigS3_BiNGO_Diagram_Clusters.pdf). Enlarged versions of the BiNGO cluster diagrams displayed in Figs. 3A-D, in which each cluster can be zoomed-in on with clarity to view all associated GO annotations and their interrelations. (Page 1) Cluster 1 (enriched in PEG); (Page 2) Cluster 2 (enriched in PEG and Unop); (Page 3) Cluster 3 (enriched in Unop); (Page 4) Cluster 4 (enriched in NC).

## Data Availability

The RNAseq datasets generated and/or analyzed during the current study are publicly available in the NCBI Gene Expression Omnibus (GEO) repository (accession #: GSE145504) (https://www.ncbi.nlm.nih.gov/geo/query/acc.cgi?acc=GSE145504). Primers for RT-qPCR were designed via the PrimerQuest tool by Integrated DNA Technologies (IDT) using complimentary mRNA transcript sequences derived from National Center for Biotechnology Information (NCBI) GenBank databases for *Rattus norvegicus* (www.ncbi.nlm.nih.gov/genbank; RRID:SCR_002760). All other data generated or analyzed during this study are included in this published article [and its supplementary information files].

## Abbreviations

CAP: Compound action potential
CMAP: Compound muscle action potential
DAMP: Damage-associated molecular pattern
DAVID: Database for Annotation, Visualization, and Integrated Discovery
DEG: Differentially expressed gene
GO: Gene ontology
IHC: Immunohistochemistry
KEGG: Kyoto Encyclopedia of Genes and Genomes
MB: Methylene blue
MHC: Major histocompatibility complex
NC: Negative control
NGS: Next-generation sequencing
PCA: Principle component analysis
PEG: Polyethylene glycol
PNA: Peripheral nerve allograft
PNI: Peripheral nerve injury
PO: Post-operatively
RNAseq: RNA sequencing
RT-qPCR: Quantitative reverse transcription PCR
TEM: Transmission electron microscopy
Unop: Unoperated control nerve
