# Supplementary material for: Coding transcriptome analyses reveal altered functions underlying immunotolerance of PEG-fused rat sciatic nerve allografts
Source: J Neuroinflammation. 2020 Oct 2;17:287. doi: 10.1186/s12974-020-01953-8 (PMC7532577; doi:10.1186/s12974-020-01953-8)
Supplement: Supplementary file 1 — Additional file 1: Fig. S1. (FigS1_Exp_Design.pdf). Outline of the experimental design of the study. PEG-fused sciatic nerve allografts (“PEG”, n=3 animals), negative control sciatic nerve allografts (“NC”, n=3 animals) from outbred Sprague Dawley rats were excised and sampled 14d post-operatively. Both treatment groups were compared to Unoperated Control sciatic nerves (“Unop”, n=2 animals). Total RNA was extracted from each sample via homogenization with TRIzol (Ambion) and a RNeasy Mini kit (Qiagen). Libraries were prepared using a TruSeq Stranded mRNA kit (Illumina), which were then sequenced on a HiSeq 3000 unit (Illumina). Analysis of read results was carried out sequentially by FastQC, Tophat2, HTSeq-count, and DESeq2 software. Subsequent analyses were performed via DAVID and the STRING application for Cytoscape software. Validation of candidate genes was performed via RT-qPCR, using the same treatment groups. [file 12974_2020_1953_MOESM1_ESM.pdf]

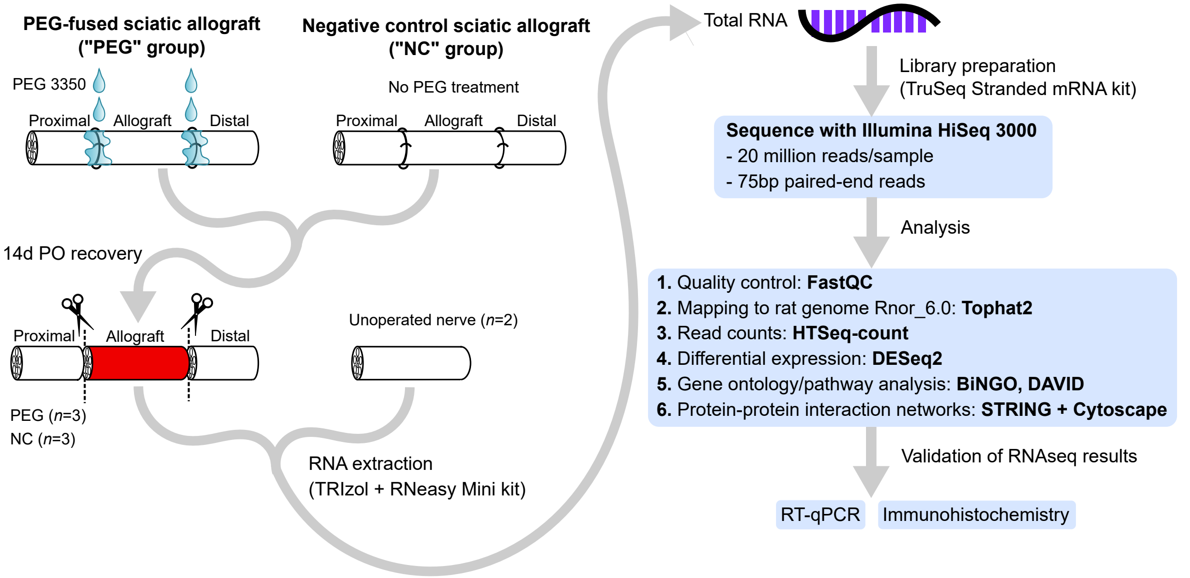
PEG-fused sciatic allograft
("PEG" group)
PEG 3350
Proximal
Allograft
Distal
Negative control sciatic allograft
("NC" group)
No PEG treatment
Proximal
Allograft
Distal
14d PO recovery
Proximal
Allograft
Distal
PEG (n=3)
NC (n=3)
Unoperated nerve (n=2)
RNA extraction
(TRIzol + RNeasy Mini kit)
Total RNA
Library preparation
(TruSeq Stranded mRNA kit)
Sequence with Illumina HiSeq 3000
- 20 million reads/sample
- 75bp paired-end reads
Analysis
1. Quality control: FastQC
2. Mapping to rat genome Rnor_6.0: Tophat2
3. Read counts: HTSeq-count
4. Differential expression: DESeq2
5. Gene ontology/pathway analysis: BiNGO, DAVID
6. Protein-protein interaction networks: STRING + Cytoscape
Validation of RNAseq results
RT-qPCR
Immunohistochemistry
