# Supplementary figures and images for: Coding transcriptome analyses reveal altered functions underlying immunotolerance of PEG-fused rat sciatic nerve allografts
Source: J Neuroinflammation. 2020 Oct 2;17:287. doi: 10.1186/s12974-020-01953-8 (PMC7532577; doi:10.1186/s12974-020-01953-8)

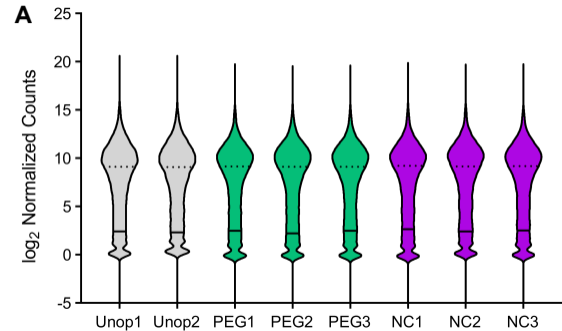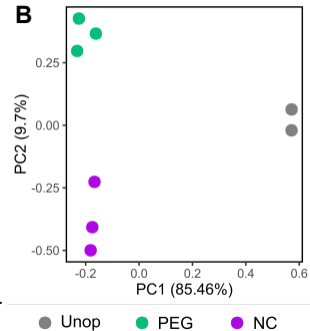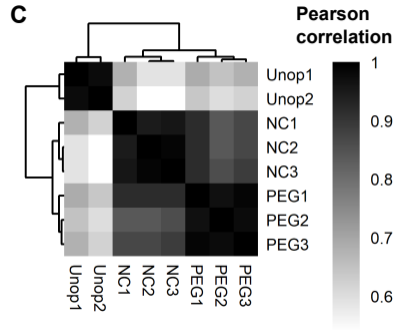

Supplement: Supplementary file 3 — Additional file 3: Fig. S2. (FigS2_Count_Analyses.pdf). Distribution and correlation of normalized read counts among samples. (A) Violin plots showing the distribution of all log2 normalized read counts for each gene transcript in each sample. The median is indicated by a solid horizontal line, and the upper quartile is indicated by a dashed horizontal line. The plots show that the majority of genes in each sample share similar expression profiles and are not differentially expressed. (B) Principle component analysis (PCA) allowing clustering of samples based on variation in expression profiles of all protein coding genes. Samples with similar expression profiles cluster together more closely. (C) Sample-to-sample correlation matrix comparing the gene expression profiles of all coding genes between all individual samples, using normalized read counts. White indicates a Pearson correlation coefficient of <0.6 (lower correlation); black indicates a coefficient of 1 (high correlation). [file 12974_2020_1953_MOESM3_ESM.pdf]
