# Supplementary material for: Coding transcriptome analyses reveal altered functions underlying immunotolerance of PEG-fused rat sciatic nerve allografts
Source: J Neuroinflammation. 2020 Oct 2;17:287. doi: 10.1186/s12974-020-01953-8 (PMC7532577; doi:10.1186/s12974-020-01953-8)

Cluster 1 - highest expression in PEG (BiNGO)
Zoom to view GO annotations

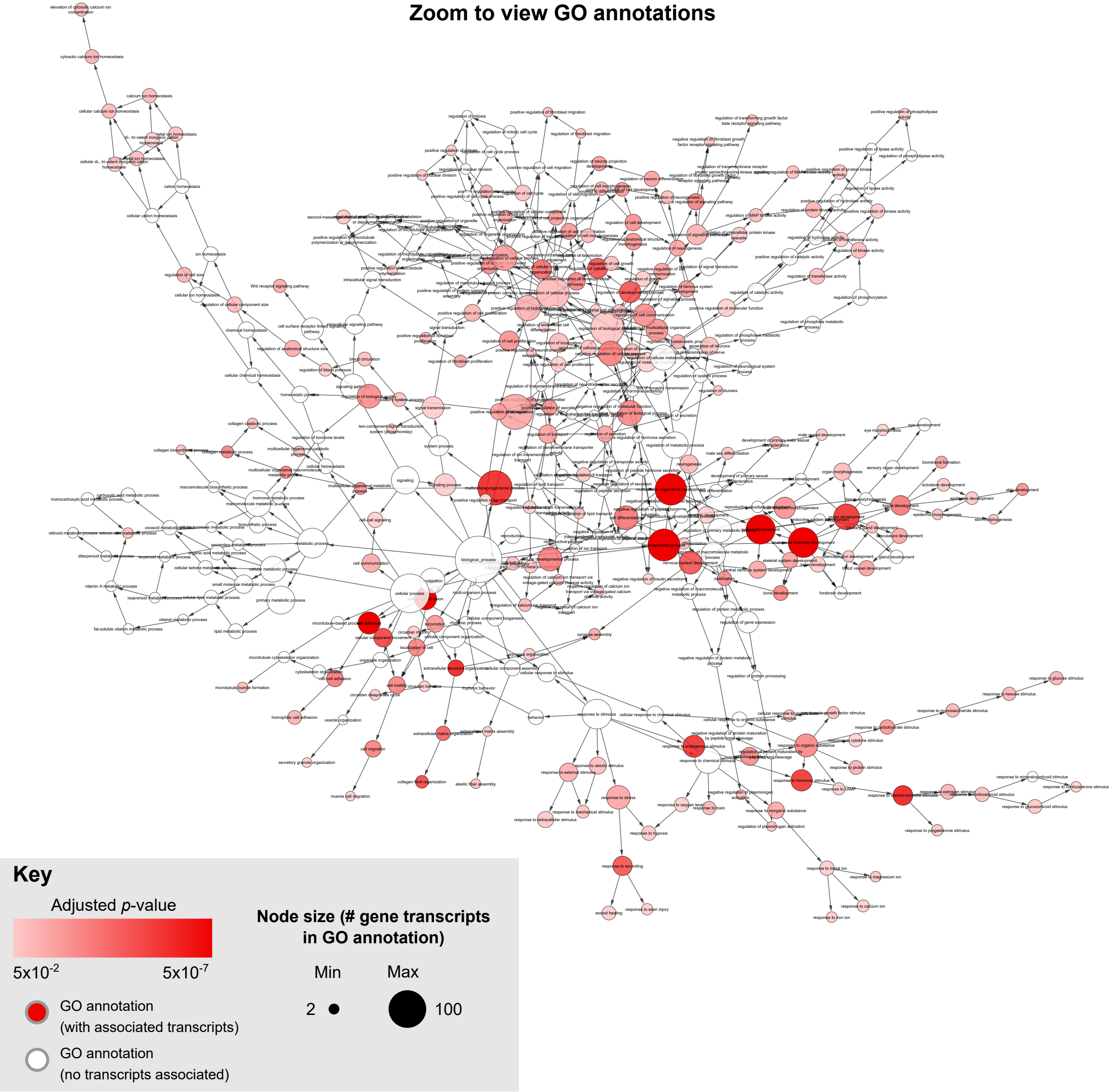

Cluster 2 - high expression in PEG and Unop (BiNGO)
Zoom to view GO annotations

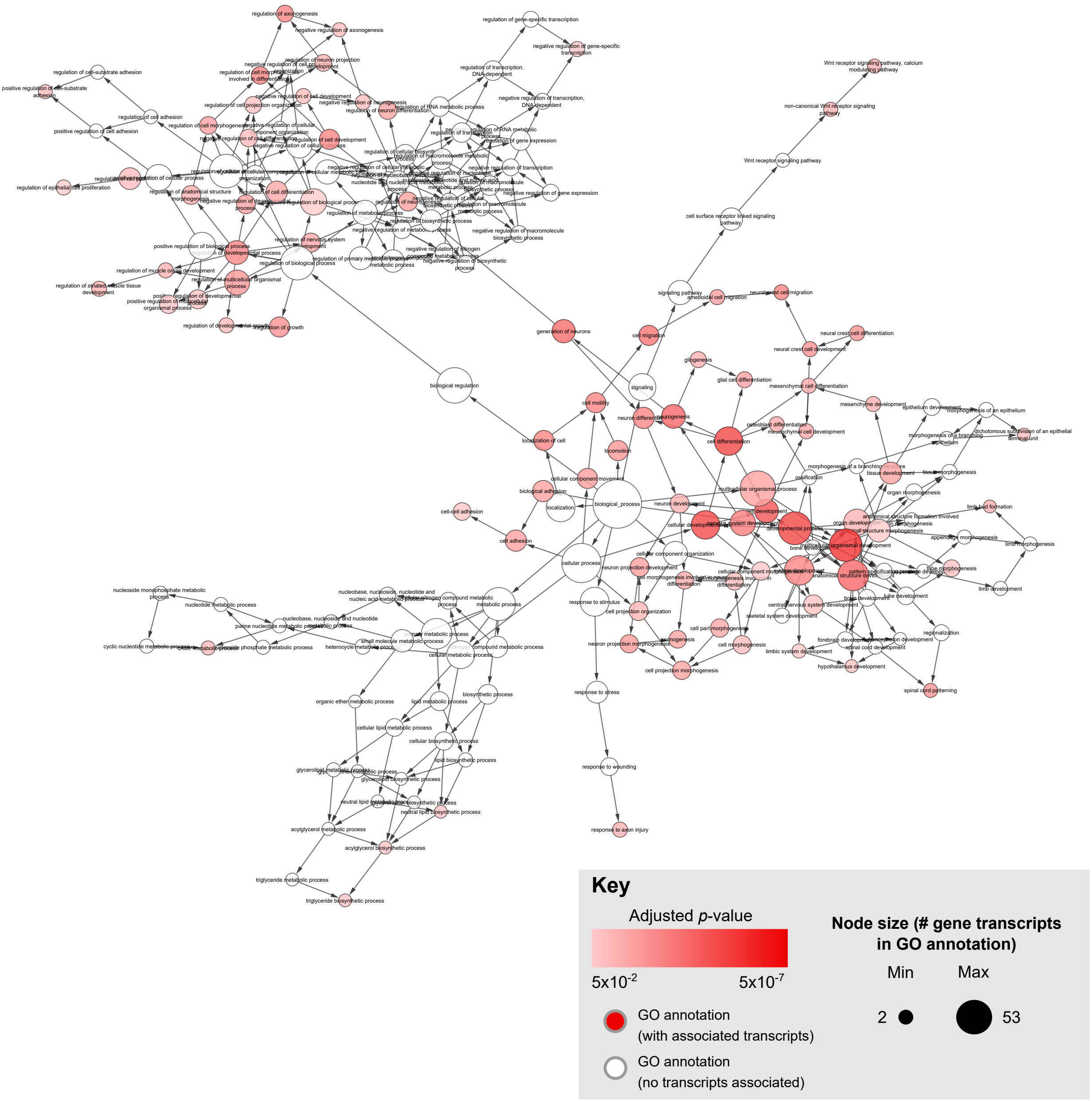

Cluster 3 - highest expression in Unop (BiNGO)
Zoom to view GO annotations

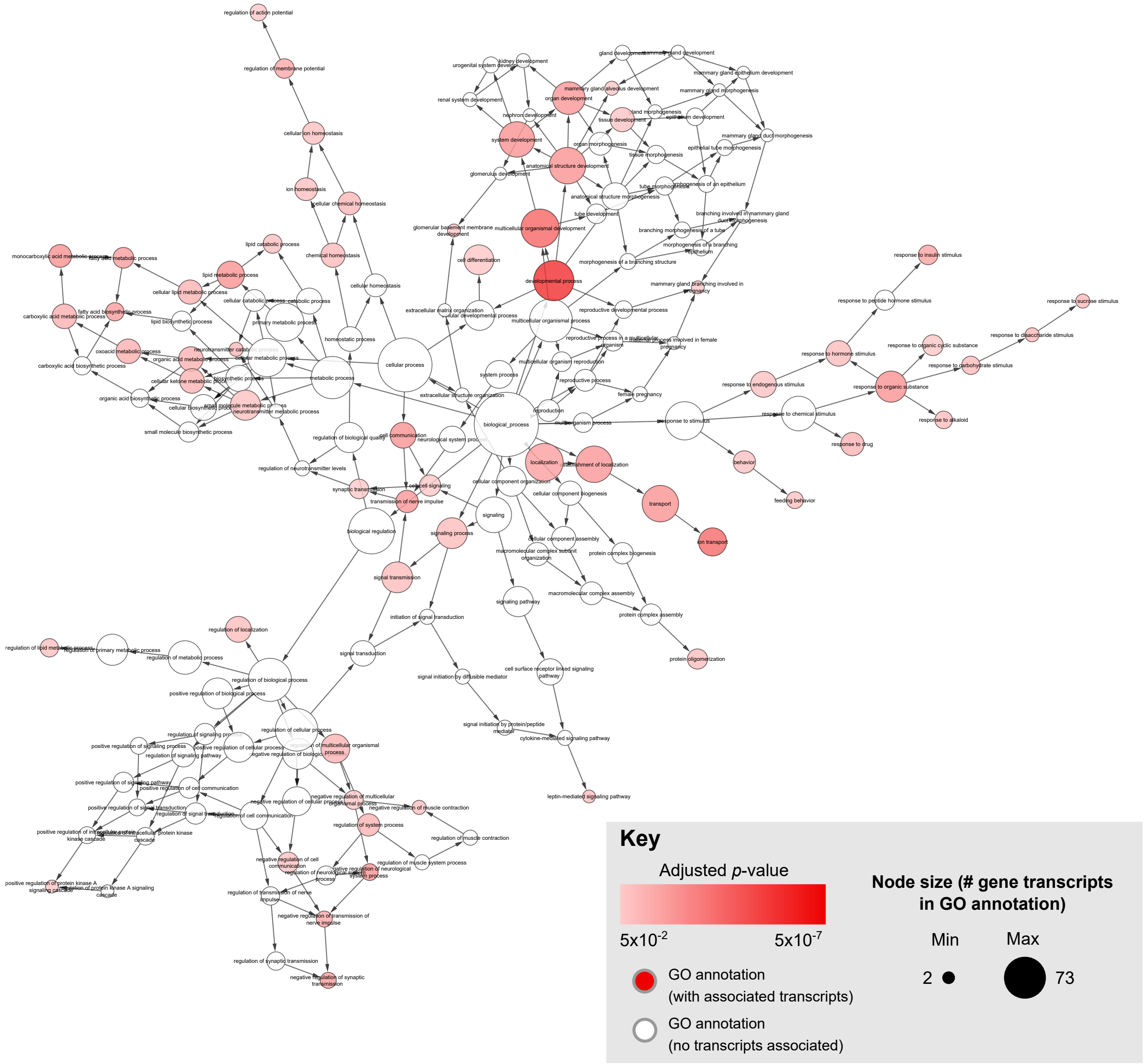

Cluster 4 - highest expression in NC (BiNGO)
Zoom to view GO annotations

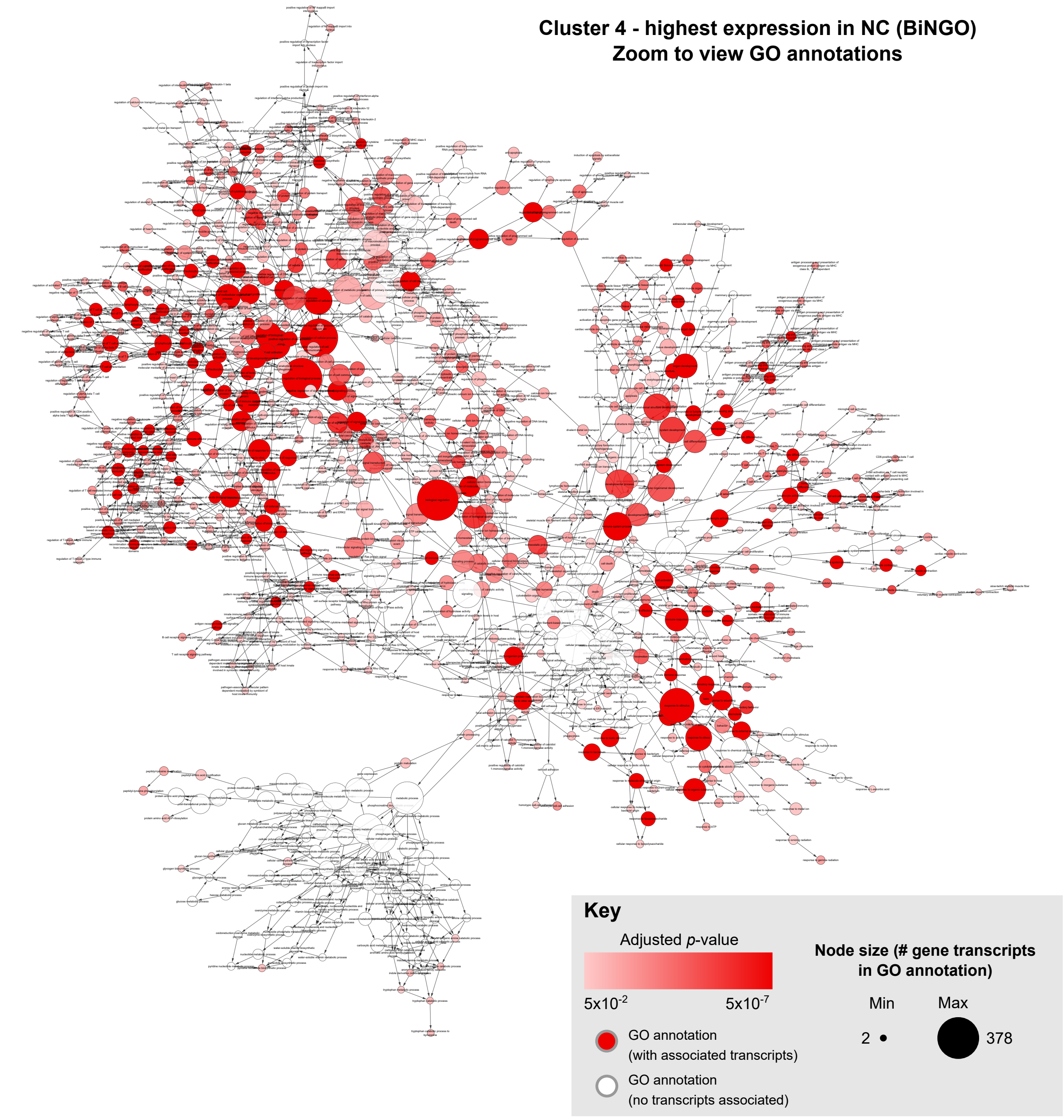

Supplement: Supplementary file 7 — Additional file 7: Fig. S3. (FigS3_BiNGO_Diagram_Clusters.pdf). Enlarged versions of the BiNGO cluster diagrams displayed in Figs. 3A-D, in which each cluster can be zoomed-in on with clarity to view all associated GO annotations and their interrelations. (Page 1) Cluster 1 (enriched in PEG); (Page 2) Cluster 2 (enriched in PEG and Unop); (Page 3) Cluster 3 (enriched in Unop); (Page 4) Cluster 4 (enriched in NC). [file 12974_2020_1953_MOESM7_ESM.pdf]
